# Genetic Associations of Type 2 Diabetes with Islet Amyloid Polypeptide Processing and Degrading Pathways in Asian Populations

**DOI:** 10.1371/journal.pone.0062378

**Published:** 2013-06-11

**Authors:** Vincent Kwok Lim Lam, Ronald Ching Wan Ma, Heung Man Lee, Cheng Hu, Kyong Soo Park, Hiroto Furuta, Ying Wang, Claudia Ha Ting Tam, Xueling Sim, Daniel Peng-Keat Ng, Jianjun Liu, Tien-Yin Wong, E. Shyong Tai, Andrew P. Morris, DIAGRAM Consortium, Nelson Leung Sang Tang, Jean Woo, Ping Chung Leung, Alice Pik Shan Kong, Risa Ozaki, Wei Ping Jia, Hong Kyu Lee, Kishio Nanjo, Gang Xu, Maggie Chor Yin Ng, Wing-Yee So, Juliana Chung Ngor Chan

**Affiliations:** 1 Department of Medicine and Therapeutics, The Chinese University of Hong Kong, The Prince of Wales Hospital, Shatin, Hong Kong SAR, People's Republic of China; 2 Department of Chemical Pathology, The Chinese University of Hong Kong, The Prince of Wales Hospital, Shatin, Hong Kong SAR, People's Republic of China; 3 Department of Orthopaedics and Traumatology, The Chinese University of Hong Kong, The Prince of Wales Hospital, Shatin, Hong Kong SAR, People's Republic of China; 4 Department of Endocrinology and Metabolism, Shanghai Diabetes Institute, Shanghai Key Laboratory of Diabetes Mellitus, Shanghai Clinical Center for Diabetes, Shanghai Key Clinical Center for Metabolic Disease, Shanghai Jiao Tong University Affiliated Sixth People's Hospital, Shanghai, People's Republic of China; 5 Department of Molecular Medicine and Biopharmaceutical Sciences, Graduate School of Convergence Science and Technology and Department of Internal Medicine, College of Medicine, Seoul National University, Chongno-gu, Seoul, Korea; 6 First Department of Medicine, Wakayama Medical University, Wakayama, Japan; 7 Hong Kong Institute of Diabetes and Obesity, The Chinese University of Hong Kong, Hong Kong SAR, People's Republic of China; 8 Li Ka Shing Institute of Health, The Chinese University of Hong Kong, The Prince of Wales Hospital, Shatin, Hong Kong SAR, People's Republic of China; 9 Centre for Molecular Epidemiology, Saw Swee Hock School of Public Health, National University of Singapore, Singapore, Singapore; 10 Saw Swee Hock School of Public Health, National University of Singapore, Singapore, Singapore; 11 Genome Institute of Singapore, Agency for Science, Technology and Research, Singapore, Singapore; 12 Singapore Eye Research Institute, Singapore National Eye Centre, Singapore, Singapore; 13 Department of Ophthalmology, Yong Loo Lin School of Medicine, National University of Singapore, Singapore, Singapore; 14 Department of Medicine, Yong Loo Lin School of Medicine, National University of Singapore, Singapore, Singapore; 15 Duke-National University of Singapore Graduate Medical School, Singapore, Singapore; 16 Wellcome Trust Centre for Human Genetics, University of Oxford, Oxford, United Kingdom; RIKEN Center for Integrated Medical Science, Japan

## Abstract

Type 2 diabetes (T2D) is a complex disease characterized by beta cell dysfunctions. Islet amyloid polypeptide (IAPP) is highly conserved and co-secreted with insulin with over 40% of autopsy cases of T2D showing islet amyloid formation due to IAPP aggregation. Dysregulation in IAPP processing, stabilization and degradation can cause excessive oligomerization with beta cell toxicity. Previous studies examining genetic associations of pathways implicated in IAPP metabolism have yielded conflicting results due to small sample size, insufficient interrogation of gene structure and gene-gene interactions. In this multi-staged study, we screened 89 tag single nucleotide polymorphisms (SNPs) in 6 candidate genes implicated in IAPP metabolism and tested for independent and joint associations with T2D and beta cell dysfunctions. Positive signals in the stage-1 were confirmed by *de novo* and *in silico* analysis in a multi-centre unrelated case-control cohort. We examined the association of significant SNPs with quantitative traits in a subset of controls and performed bioinformatics and relevant functional analyses. Amongst the tag SNPs, rs1583645 in carboxypeptidase E (*CPE*) and rs6583813 in insulin degrading enzyme (*IDE*) were associated with 1.09 to 1.28 fold increased risk of T2D (*P*_Meta_ = 9.4×10^−3^ and 0.02 respectively) in a meta-analysis of East Asians. Using genetic risk scores (GRS) with each risk variant scoring 1, subjects with GRS≥3 (8.2% of the cohort) had 56% higher risk of T2D than those with GRS = 0 (*P* = 0.01). In a subcohort of control subjects, plasma IAPP increased and beta cell function index declined with GRS (*P* = 0.008 and 0.03 respectively). Bioinformatics and functional analyses of *CPE* rs1583645 predicted regulatory elements for chromatin modification and transcription factors, suggesting differential DNA-protein interactions and gene expression. Taken together, these results support the importance of dysregulation of IAPP metabolism in T2D in East Asians.

## Introduction

Type 2 diabetes (T2D) is characterized by abnormal beta cell biology. Large scale genome-wide association studies (GWAS) have discovered multiple loci associated with T2D in both European [1] and Asian populations [2]. While some of these risk variants independently conferred 1.1–1.5 fold increased risk, this could increase to 2–3 folds in carriers with multiple genetic variants [3]. Islet amyloid polypeptide (IAPP) is highly conserved and co-secreted with insulin with suppressing effects on appetite [4]. Over 40% of T2D autopsy cases in human showed amyloid deposits associated with loss of beta cells [5]. IAPP is synthesized as a prohormone (pro-IAPP) which is processed to mature IAPP in endoplasmic reticulum (ER) by several enzymes and proteins, including prohormone convertases (PCSK1, PCSK2), carboxypeptidase E (CPE) and serum amyloid P component (APCS) before cleared by the insulin-degrading enzyme (IDE). Dysregulation of these processing enzymes, increased stabilization of IAPP by APCS and reduced clearance of IAPP by IDE [6], [7], [8] can lead to accumulation of pro-IAPP or excessive oligomerization of IAPP [9] which can cause mitochondrial dysfunction [10] and ER stress [11]. Excessive pro-IAPP and IAPP production can also lead to formation of amyloid beta sheet resulting in loss of islet structure and beta cell function [9] (Figure S1).

Research studies including GWAS have revealed independent associations of T2D with genetic polymorphisms of components of IAPP metabolism [12], [13], [14]. However, these results were not always consistent [15], [16] due to small sample size, population heterogeneity and incomplete interrogation of gene structure. The *hematopoietically expressed homeobox (HHEX)*-*IDE* block is one of the GWAS susceptibility loci for T2D with replications in multiple ethnic groups [3], [14]. Although *HHEX* is considered to be the most likely causal gene in this block, some studies had shown independent effect of genetic polymorphisms of *IDE* and their combined effects with *HHEX* on risk of T2D [17].

**Figure 1:**
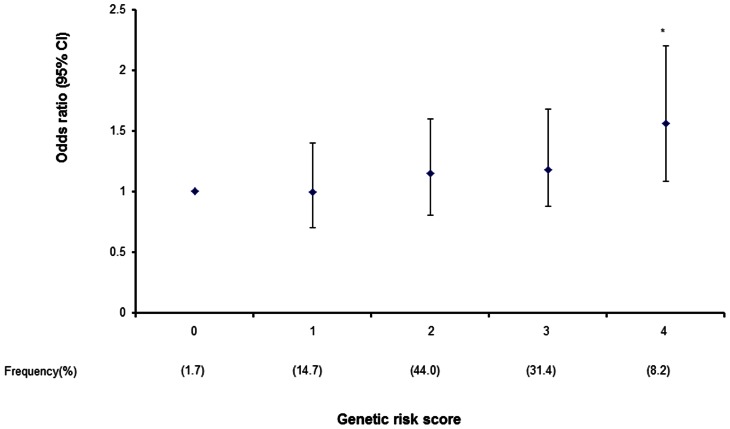
Based on results of a meta-analysis of risk association of type 2 diabetes (T2D) in 9,901 Asian subjects with *de novo* genotyping, each risk allele of rs1583645 (*CPE*) and rs6583813 (*IDE*) was given a genetic risk scores (GRS) of 1 under additive models. Increasing GRS was associated with increasing trend of risk for T2D (*P*_meta_  = 0.01; Q-statistic *P*<0.05) with the highest GRS of 4 conferring an odds ratios of 1.56 compared to the lowest GRS of 0 (*P = 0.01).

**Figure 2:**
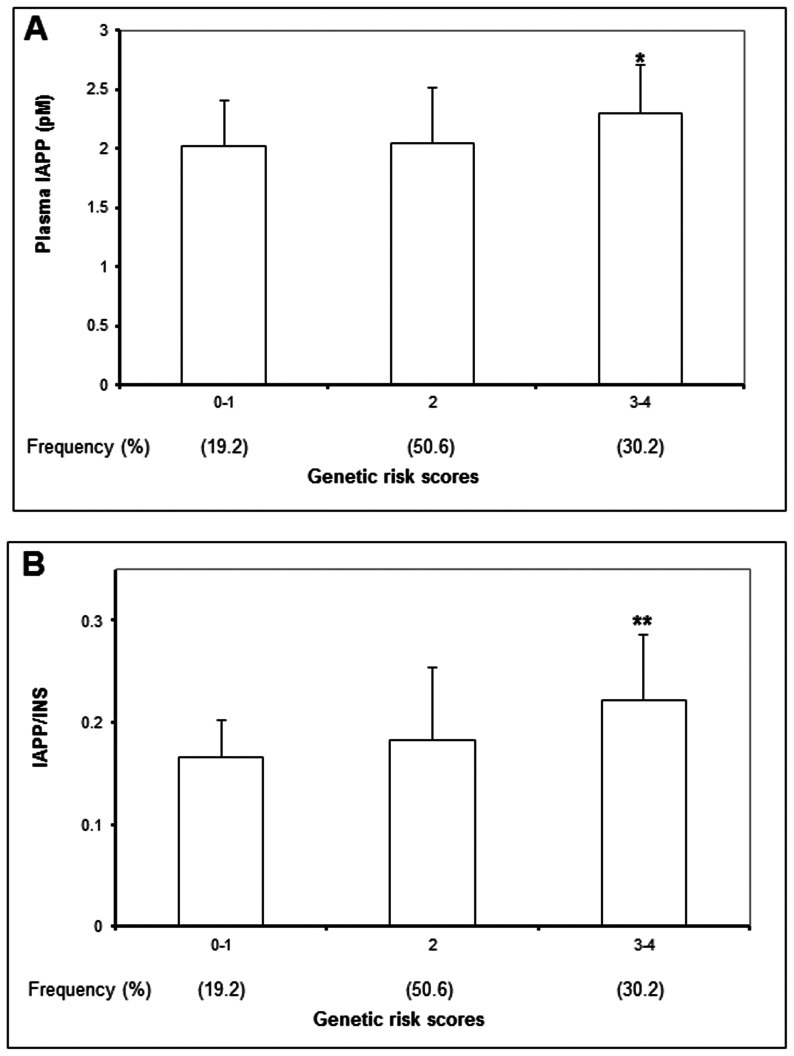
Plasma IAPP (A) and molar ratio of IAPP to insulin (IAPP/INS) (B) in 85 unrelated non-diabetic controls selected from a family-based cohort categorized by genetic risk scores (GRS) (1 risk allele of rs1583645 of *CPE* and rs6583813 of *IDE* each given 1 point). Plasma IAPP (*P = 0.008) and IAPP/INS ratio (**P = 0.006) increased with increasing GRS.

**Figure 3:**
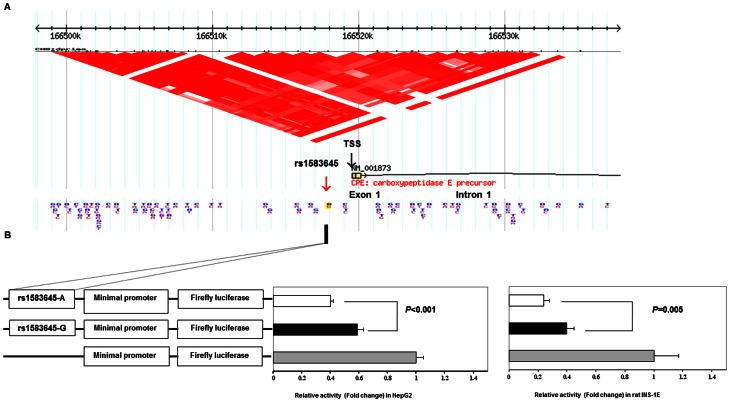
Effect of rs1583645 [G/A] polymorphism on luciferase activity assays. (A) Upstream region of transcription start site (TSS as indicated by the black arrow), first exon and part of intron 1 of *CPE* (NCBI Build 36.1, CHR4:166,496,501–166,536,501). The LD structure of *CPE* SNPs within this region was shown by D' using the Chinese HapMap data. The red arrow indicated the location of rs1583645. (B) *CPE*-[G/A] constructs consisting of 449 bp of *CPE* rs1583645 region and pGL4.23 firefly luciferase reporter vectors were transfected into HepG2 (left panel) and rat INS-1E cells (right panel) together with *Renilla* luciferase reporter vectors. Measurement of the firefly luciferase activity of *CPE*-[G/A] constructs was normalized relative to the activity of the *Renilla* luciferase vectors. Data were shown as mean±SEM of at least three independent experiments in triplicate set up. The constructs of *CPE*-G showed 50% and 66.7% increased transcriptional activity in HepG2 and rat INS-1E cells respectively when compared to the constructs of *CPE*-A (*P*<0.001 and *P* = 0.005 respectively by Mann-Whitney *U*-test).

In this report, we used a tag single nucleotide polymorphism (SNP) approach to select genetic variants of candidate genes (*APCS*, *CPE*, *IAPP, IDE, PCSK1* and *PCSK2*) implicated in IAPP metabolism (Figure S1) and tested their independent and joint associations with risk of T2D and beta cell dysfunction. In this multi-staged experiment, we performed *de novo* genotyping in 9,901 Asians and *in silico* analysis in 55,252 subjects followed by bioinformatics and functional analyses (Table 1–3 and Figure 1–3). The study design was summarized in Figure S2.

**Table 1:** Association of type 2 diabetes (T2D) with risk variants of carboxypeptidase E (*CPE)* and insulin degrading enzyme (*IDE)* in a multi-staged experiment using a tag SNP approach applied to discovery cohort in Hong Kong Chinese (Stage-1) followed by *de novo* genotyping of top signals in a multi-ethnic Asian population.

		N		Genotypes		Allelic		Recessive		Dominant		Stage-1 and 2 combined^c^		
	Risk					*P*	Odds	*P*	Odds	*P*	Odds	*P*_Meta_	Odds	Cochran's
	Allele	T2D	Controls	T2D	Controls	values	ratios	values	ratios	values	ratios	values	ratios _Meta_	Q statistic
CPE														
rs1583645	G			AA/GA/GG	AA/GA/GG									
Stage-1														
Hong Kong Chinese		410	386	18/153/239	30/151/205	**0.05^b^**	**1.26(1.0**–**1.57)**	0.141	1.23(0.93–1.63)	**0.045**	**1.84(1.01**–**3.32)**	**0.001**	**1.16(1.06**–**1.26)**	0.067
Stage-2														
Hong Kong Chinese		1079	1969	45/368/666	110/740/1119	**0.005**	**1.2(1.05**–**1.36)**	**0.009**	**1.22(1.05**–**1.43)**	0.089	1.36(0.95–1.94)			
Shanghai Chinese		1618	1634	44/361/1213	37/446/1151	**0.021**	**1.17(1.02**–**1.35)**	**0.004**	**1.26(1.08**–**1.47)**	0.405	0.83(0.53–1.29)			
Japanese		568	582	15/126/427	11/138/433	0.993	1(0.79–1.27)	0.762	1.04(0.8–1.36)	0.392	0.71(0.32–1.55)			
Korean		754	629	20/191/543	13/143/473	0.161	0.86(0.69–1.06)	0.182	0.85(0.67–1.08)	0.477	0.77(0.38–1.57)			
rs6841638	G			TT/GT/GG	TT/GT/GG									
Stage-1														
Hong Kong Chinese		428	416	31/125/272	27/166/223	**0.04^b^**	**1.3(1.04**–**1.62)**	**0.003**	**1.51(1.15**–**1.99)**	0.666	0.89(0.52–1.52)	0.51	1.06(0.9–1.24)	0.014
Stage-2														
Hong Kong Chinese		1081	1968	47/372/662	100/648/1220	0.993	1(0.88–1.14)	0.683	0.97(0.83–1.13)	0.366	1.18(0.83–1.68)			
Shanghai Chinese		1688	1664	56/551/1081	58/493/1113	0.162	0.92(0.81–1.04)	0.083	0.88(0.76–1.02)	0.788	1.05(0.72–1.53)			
Japanese		568	582	15/142/411	17/150/415	0.659	1.05(0.84–1.32)	0.691	1.05(0.81–1.36)	0.773	1.11(0.55–2.24)			
Korean		757	632	20/181/556	15/165/452	0.542	1.07(0.87–1.32)	0.422	1.1(0.87–1.4)	0.750	0.9(0.45–1.76)			
rs10021007	C			AA/CA/CC	AA/CA/CC									
Stage-1														
Hong Kong Chinese		429	415	48/185/196	62/197/156	**0.01^b^**	**1.30(1.06**–**1.58)**	**0.017**	**1.4(1.06**–**1.84)**	0.106	1.39(0.93–2.08)	0.61^d^	1.07(0.83–1.34)^d^	0.03^d^
Stage-2														
Hong Kong Chinese		1069	1978	114/489/466	247/908/823	0.136	1.09(0.97–1.22)	0.290	1.08(0.93–1.26)	0.137	1.2(0.94–1.51)			
Shanghai Chinese		1216	1577	103/468/645	147/655/775	0.053	1.12(1–1.26)	**0.041**	**1.17(1.01**–**1.36)**	0.435	1.11(0.85–1.45)			
Japanese		568	582	41/193/334	21/199/362	**0.044**	**0.82(0.67**–**0.99)**	0.239	0.87(0.68–1.1)	**0.007**	**0.48(0.28**–**0.82)**			
Korean		754	629	46/288/420	46/257/326	0.131	1.14(0.96–1.35)	0.150	1.17(0.95–1.45)	0.368	1.21(0.8–1.85)			
rs17046561	G			AA/GA/GG	AA/GA/GG									
Stage-1														
Hong Kong Chinese		423	412	6/100/317	10/119/283	**0.03^b^**	**1.34(1.02**–**1.75)**	**0.045**	**1.36(1.01**–**1.84)**	0.288	1.73(0.63–4.74)	0.25^e^	1.05(0.96–1.15)^e^	0.16^e^
Stage-2														
Hong Kong Chinese		1080	1978	23/278/779	62/512/1404	0.269	1.09(0.94–1.26)	0.502	1.06(0.9–1.25)	0.106	1.49(0.92–2.41)			
Shanghai Chinese		1618	1649	17/294/1307	20/278/1351	0.504	0.95(0.8–1.11)	0.399	0.93(0.78–1.11)	0.661	1.16(0.6–2.21)			
Japanese		568	582	2/98/468	3/116/463	0.224	1.19(0.9–1.56)	0.220	1.2(0.9–1.62)	0.674	1.47(0.25–8.71)			
Korean		752	630	3/125/624	4/92/534	0.464	0.9(0.69–1.19)	0.370	0.88(0.66–1.17)	0.538	1.6(0.36–7.06)			
*IDE*														
rs6583813^a^	C			CC/CT/TT	CC/CT/TT									
Stage-1														
Hong Kong Chinese		429	414	62/188/179	47/167/200	**0.04^b^**	**1.24(1.01**–**1.51)**	0.18	1.32(0.88–1.98)	0.055	1.31(0.99–1.71)	**0.045**	**1.35(1.01**–**1.81)**	<0.001
Stage-2														
Hong Kong Chinese		1076	1952	128/480/468	252/847/853	0.751	0.98(0.88–1.1)	0.420	0.91(0.73–1.14)	0.914	1.01(0.87–1.17)			
Shanghai Chinese		1292	1576	153/449/690	164/608/804	0.693	0.98(0.87–1.1)	0.222	1.16(0.92–1.46)	0.202	1.1(0.95–1.28)			
Japanese		568	582	93/267/208	48/249/285	**<0.001**	**1.57(1.33**–**1.87)**	**<0.001**	**2.18(1.51**–**3.13)**	**<0.001**	**1.66(1.31**–**2.1)**			
Korean		756	630	128/349/279	70/294/266	**0.003**	**1.27(1.09**–**1.48)**	**0.002**	**1.63(1.2**–**2.22)**	**0.044**	**1.25(1.01**–**1.55)**			
*PCSK2*														
rs8117664	G			GG/GT/TT	GG/GT/TT									
Stage-1														
Hong Kong Chinese		424	416	16/119/289	7/102/307	**0.03^b^**	**1.32(1.02**–**1.72)**	0.063	2.29(0.96–5.5)	0.072	1.32(0.98–1.77)	0.35^e^	0.96(0.89–1.04)^e^	0.108^e^
Stage-2														
Hong Kong Chinese		1075	1978	28/272/775	74/524/1380	0.081	0.88(0.76–1.02)	0.095	0.69(0.44–1.07)	0.178	0.89(0.76–1.05)			
Shanghai Chinese		1161	1542	56/339/766	65/485/992	0.635	0.97(0.85–1.11)	0.449	1.15(0.8–1.66)	0.375	0.93(0.79–1.09)			
Japanese		568	582	21/174/373	16/202/364	0.510	0.93(0.76–1.15)	0.362	1.36(0.7–2.62)	0.269	0.87(0.69–1.11)			
Korean		749	627	26/262/461	24/221/382	0.754	0.97(0.81–1.17)	0.725	0.9(0.51–1.59)	0.813	0.97(0.78–1.21)			

a rs2149632 in high LD with rs6583813 (r^2^ =  0.94; D' = 1) was genotyped in Shanghai Chinese.

*P* or ^b^ *P_empirical_* values and ORs with nominal significance for T2D risk (*P*≤0.05) were shown in bold.

c The meta-analysis among five unrelated case-control cohorts (Stage-1 study: Hong Kong Chinese; Stage-2 study: Additional Hong Kong Chinese, Shanghai Chinese, Japanese and Korean) was performed in the best genetic model by the fixed effects of Cochran-Mantel-Haenszel (CMH) test. Heterogeneity of ORs among studies was assessed by Cochran's Q statistics .The effect size calculated from the random effects model if Q-statistic *P* was smaller than 0.05.

d and ^e^ indicated the meta-analysis conducted using dominant and allelic models respectively otherwise was recessive model.

**Table 2:** Associations of genetic risk scores (GRS) with beta cell function in Hong Kong Chinese unrelated controls (N = 419) with 1 risk allele of rs1583645 of *CPE* and rs6583813 of *IDE* each given 1 point.

*Genetic risk score (GRS)*	0-1	2	3–4	P value
Subjects (%)	30	41	29	
Male (%)	42	38	36	
Age (years)	41±10	40±11	40±10	
Body mass index (kg/m^2^)	22.5±3.2	22.6±3.3	22.5±3.1	
***Results of 75g oral glucose tolerance test***				
Fasting plasma glucose (mmol/l)^ a^	4.8 (4.6,5)	4.72 (4.45,5.1)	4.8 (4.6,5.1)	0.88
Fasting plasma insulin (pmol/l)^a^	41.4 (26,60.7)	41.5 (25.2,54.1)	37.3 (24.1,56.7)	0.22
Plasma glucose at 30-minute (mmol/L)^a^	7.67 (6.68,8.75)	7.78 (6.85,8.87)	7.86 (6.89,8.61)	0.31
Plasma insulin at 30-minute (pmol/L)^a^	286 (180,449)	292 (182,427)	288 (196,407)	0.24
Glucose AUC at 30-minute (min.mmol/l)^a^	195 (179,205)	190 (175,207)	191 (177,210)	0.73
Insulin AUC at 30-minute (min.pmol/l)^a^	5817 (3583,7811)	5670 (3555,8432)	4856 (3574,6922)	**0.05**
Stumvoll's index of beta cell function (×10^−6^)^a^	29.7 (21.7,40.7)	30.8 (19.8,42.8)	27.1 (18.4,36.1)	**0.03**

Data were shown as mean±SD or **^a^**median(interquartile range) and analyzed by the linear regression with adjustment of age, sex and BMI under additive models after log-transformation. *P* values in bold indicated significance for the phenotypes. AUC: area under the curve.

**Table 3:** Meta-analysis of risk associations of *CPE* rs1583645 and *IDE* rs6583813 with Type 2 diabetes (T2D) using data from *de novo* genotyping and *in silico* analysis in a multi-ethnic population.

							Risk Allele			
		CHR:bp in			Number		Frequency			
SNP	Gene	NCBI Build 36.1	Alleles^a^	Study	Cases	Controls	Cases	Controls	OR _trend_(95% CI)	*P*_trend_
rs1583645	*CPE*	CHR4:166,517,901	G/A	Stage-1						
				Hong Kong Chinese	410	386	0.770	0.727	1.26(1–1.58)	0.049
				Stage-2 *denovo* replication						
				Hong Kong Chinese	1079	1969	0.788	0.756	1.20(1.06–1.36)	5.24×10^−3^
				Shanghai Chinese	1618	1634	0.861	0.841	1.18(1.02–1.35)	0.021
				Korean	754	629	0.847	0.866	0.86(0.69–1.06)	0.161
				Japanese	568	582	0.873	0.863	1.00(0.79–1.27)	0.993
				Stage-3 *in silico* analysis						
				Singapore Chinese	2009	1945	0.800	0.799	1.00(0.89–1.12)	1.00
				Singapore Malay	1235	792	0.65	0.62	0.88(0.77–1.00)	0.06
				Singapore Indian	1166	971	0.66	0.65	0.96(0.84–1.09)	0.52
				DIAGRAM+	38987	8130	^b^0.51	–	1.00(0.96–1.04)	0.92
				^c^Meta-analysis in Asian subjects						
				Fixed effect					1.09(1.02–1.16)	9.4×10^−3^
				Random effect					1.01(0.85–1.2)	0.898
				Heterogeneity test						*P* = 0.05
rs6583813	*IDE*	CHR10:94,199,919	C/T	Stage-1						
				Hong Kong Chinese	429	414	0.364	0.315	1.23(1.01–1.49)	0.042
				Stage-2 *denovo* replication						
				Hong Kong Chinese	1076	1952	0.342	0.346	0.98(0.88–1.1)	0.754
				Shanghai Chinese	1292	1576	0.292	0.297	0.98(0.88–1.09)	0.708
				Korean	756	630	0.40	0.344	1.27(1.08–1.48)	3.0×10^−3^
				Japanese	568	582	0.399	0.296	1.58(1.32–1.88)	3.43×10^−7^
				Stage-3 *in silico* analysis						
				Singapore Chinese	1935	1879	0.315	0.278	1.20(1.09–1.33)	4.0×10^−4^
				Singapore Malay	1188	759	0.28	0.30	1.07(0.93–1.24)	0.34
				Singapore Indian	–	–	–	–	–	–
				DIAGRAM+	38987	8130	^b^0.68	–	1.17(1.12–1.22)	1.33×10^−12^
				^c^Meta-analysis in Asian subjects						
				Fixed effect					1.23(1.14–1.34)	8.25×10^−7^
				Random effect					1.28(1.04–1.59)	0.02
				Heterogeneity test						*P* = 0.002

a Risk alleles were underlined.^ b^The allele frequency was based on HapMap Caucasian (CEU) population. ^c^Meta-analysis for the Chinese from Hong Kong, Shanghai.

and Singapore, Korean and Japanese cohorts.

## Results

Table S1 shows clinical characteristics of the study populations. In the stage-1 study, 459 unrelated Chinese T2D patients and 419 age and sex-matched controls were included. Positive signals were replicated in 3,092 Hong Kong Chinese (1,114 cases and 1,978 controls), 3,388 Shanghai Chinese (1,716 cases and 1,672 controls), 1,393 Korean (761 cases and 632 controls) and 1,150 Japanese (568 cases and 582 controls). The study cohorts of Singaporeans and Europeans in *in silico* analysis were described in Table S2.

We also examined the risk association of T2D in a family-based cohort of Hong Kong Chinese consisting of 285 subjects with diabetes and 187 without diabetes. In a subset of 85 control subjects in whom IAPP and insulin were measured, we examined the risk association of significant SNPs with beta cell function. In the control subjects from the stage-1 study, we examined the association of positive SNPs with quantitative traits function.

### Stage-1 study

In the stage-1 study, we genotyped 89 single nucleotide polymorphisms (SNPs) of the 6 target genes in 878 unrelated cases and controls. Based on the HapMap Chinese data (CHB), 542 common SNPs in these genes were filtered for the tag SNP selection. In addition to 7 reported SNPs, 135 SNPs were included in the panel design with 89 SNPs finally selected for multiplex genotyping with an average call rate of 95% and concordance rate of 99.9% among the duplicate samples. Using these 89 SNPs, we were able to capture 426 common SNPs, i.e. 79% of all common SNPs with minor allele frequency (MAF) ≥0.05. Amongst these SNPs, 4 SNPs [rs2808661 (*APCS*), rs12306305 and rs1056007 (*IAPP*), and rs4646953 (*IDE*)] failed quality control (QC) and were excluded for analysis. All SNPs were in Hardy-Weinberg equilibrium (HWE) (*P*>0.001 for controls). Table S3 shows the *P_allelic_* and *P_empirical_* values for T2D of all SNPs, the latter generated by 10,000 permutations under the best model of genetic models for multiple test correction. Six of these SNPs showed nominal associations with 4 SNPs (rs1583645, rs6841638, rs10021007 and rs17046561) in *CPE*, 1 SNP (rs6583813) in *IDE* and 1 SNP (rs8117664) in *PCSK2* with odds ratio (OR) and 95% confidence intervals (CI) ranging from 1.24 (1.01–1.51) to 1.34 (1.02–1.75)(*P_empirical_* = 0.01–0.05).

### Stage-2 de novo genotyping

We replicated these 6 SNPs in 9,023 Asian subjects from Hong Kong, Shanghai, Japan and Korea, with over 90% power to detect ORs ranging from 1.24 to 1.34 at the 5% significant level. In the Shanghai Chinese, rs6583813 was discarded due to unsuccessful panel optimization and replaced by rs2149632 in a linkage disequilibrium (LD) block with rs6583813 [r^2^ = 0.94; D' = 1; MAF = 0.35 for both SNPs using HapMap CHB data]. Table 1 summarizes the results in each case-control cohort and meta-analysis of stage-1 and 2 studies under allelic, dominant and recessive models for each associated SNP. There were nominal associations of T2D with rs1583645 and rs10021007 of *CPE* and rs6583813 of *IDE* in at least one genetic model with some population heterogeneity possibly due to sub-ethnicity and other disease modifiers. For each SNP, we selected the most significant genetic model and applied a fixed effect model for SNPs which did not show heterogeneity of ORs (Q-statistic *P*>0.05). Otherwise, a random effect model was used. In the combined analysis, rs1583645 of *CPE* and rs6583813 of *IDE* were nominally associated with T2D (*P*_Meta_  = 0.001 and 0.045 respectively).

### Joint effects of CPE and IDE genetic polymorphisms

We tested the joint effects of rs1583645 in *CPE* and rs6583813 in *IDE* on T2D risk in the Asian case-control cohort and beta cell function in a subset of the Hong Kong Chinese controls. We assigned each risk allele of rs1583645 (*CPE*) and rs6583813 (*IDE*) as a genetic risk score (GRS) of 1 under additive models, which was linearly associated with T2D risk (*P*_meta_ = 0.01, Q-statistic *P*<0.05 Figure 1) on meta-analysis. Subjects with the highest GRS accounted for 8.2% of the study population and had 56% higher risk for T2D compared to those with the lowest GRS (*P* = 0.01). In the control subjects of the Hong Kong cohort stratified by GRS≤1, 2 and ≥3 with similar numbers in each group, increasing GRS was associated with progressive decline in Stumvoll's index of beta cell function (*P* = 0.03) and area under the curve (AUC) of insulin at 30-minute (*P* = 0.05, Table 2). In the 472 subjects from the Hong Kong family-based cohort, 85 non-diabetic unrelated subjects had measurement of plasma IAPP levels. In these subjects, the GRS was associated with increased plasma IAPP (*P* = 0.008) and IAPP to insulin (IAPP:INS) molar ratios (*P* = 0.006) after adjustment for age, sex, BMI and/or fasting insulin (Figure 2A and 2B).

### In silico analysis in T2D GWAS studies and combined meta-analysis

Having discovered and replicated the risk association of rs1583645 of *CPE* and rs6583813 of *IDE* SNPs with T2D in stage-1 and stage-2 experiments, we performed *in silico* analysis to validate these findings in 2 GWAS. The Singapore cohort consisted of 8,135 (3,781 cases and 4,354 controls) and the European cohort, 47,117 subjects (8,130 cases and 38,987 controls) [18], [19] (Table 3). In the Asian (CHB+JPK) and European (CEU) HapMap database, the respective frequency of the G allele of rs1583645 of *CPE* were 0.87 and 0.51 while that of the C allele of rs6583813 of *IDE* were 0.39 and 0.68 (Table S4). In the Caucasian population, we found strong association of *IDE* rs6583813 with T2D (*P* = 1.33×10^−12^) but not with rs1583645 of *CPE.* Due to this inter-ethnic differences, we only included *de novo* genotyping and *in silico* analysis of GWAS data from the East Asian populations and confirmed the risk association for *CPE* rs1583645 [OR:1.09(1.02–1.16), *P* = 9.4×10^−3^] in the fixed effect model and *IDE* rs6583813 [OR:1.28(1.04–1.59), *P* = 0.02] in the random effect model.

### Bioinformatics and functional analyses

Both *CPE* and *IDE* loci show mild conservation among species and are located in the vicinity of regulatory elements for histone modification, islet specific formaldehyde-assisted isolation of regulatory elements (FAIRE) and DNaseI hypersensitive (HS) peaks related to open chromatin modifications. A downstream region from *CPE* rs1583645 is annotated with a CpG island and pre-microRNA form (Table S5). Using transcription factor binding site (TFBS) prediction tools, we identified 11 transcription factors (TFs) which can either bind specifically to one or both of these variants (Table S6). Due to its proximity to the promoter region, we conducted functional tests on *CPE* rs1583645 variants [G/A] using dual luciferase reporter assays transiently transfected in HepG2 and rat INS-1E cells. In both cells transfected with the constructs carrying the G-risk allele, the basal luciferase activity was 50–66.7% higher than those transfected with A-allele of rs1583645 [*P*<0.001 and *P* = 0.005 respectively, Mann-Whitney *U*-test (Figure 3)].

## Discussion

In this multi-stage study, we used a hypothesis defined *a priori* and a tag SNP approach combined with genetic statistics, bioinformatics and functional analyses to examine the independent and joint effects of components of the IAPP pathway on risk of T2D and beta cell function. In the meta-analysis of *de novo* genotyping and *in silico* analysis of GWAS data in Asian populations, we confirmed the risk association of rs1583645 in *CPE* and rs6583813 in *IDE* with T2D. Using GRS, we demonstrated the joint effects of these two variants with increased plasma IAPP and reduced beta cell function. These findings were corroborated by bioinformatics analysis suggesting that the flanking region of these SNPs might harbor regulatory elements for gene expression through chromatin modification and binding with TFs [20], [21]. Results of luciferase activity assays indicated the G risk allele was associated with lower *CPE* repression than the non-risk allele which might cause dysregulation of IAPP production and beta cell dysfunction.

In the genetic analysis, we used data from the HapMap Project and selected tag SNPs which captured over 80% of common SNPs with MAF≥5% for each of these 6 candidate genes. The *de novo* genotyping of unrelated case-control cohort consisting of 9,901 subjects had over 90% power to detect at least 20% increased risk for T2D for SNPs with MAF of 10%. In the first stage experiment, we selected 6 SNPs in *CPE*, *IDE* and *PCSK2* which showed nominal significance for replication. Notwithstanding some heterogeneity of effect sizes possibly due to differences in population-specific LD architecture, allele frequency [18] and factors such as lifestyle and environment [22], rs1583645 of *CPE* and rs6583813 of *IDE* showed consistent associations with T2D on meta-analysis of multiple East Asian cohorts. Importantly, subjects with ≥3 risk variants, which accounted for 8.2% of the study population, had 56% increased risk of T2D. In the control subjects, increased GRS was associated with increased plasma IAPP and reduced insulin secretion. Taken together, these findings support our hypothesis that dysregulation of IAPP pathway might increase risk of beta cell dysfunction and T2D.

### Insulin degrading enzyme (IDE)

Both *CPE* and *IDE* are widely expressed to process and degrade different hormones including IAPP and insulin. In experimental studies, inhibition of *IDE* decreased IAPP degradation and increased IAPP toxicity while *CPE* mediated palmitate-induced ER stress resulting in beta cell apoptosis [7], [23], [24]. These findings were supported by association of variants of *CPE* and *IDE* with risk of T2D and related traits in small cohort studies [25], [26]. In another study, rs2149632 of *IDE* was associated with reduced insulin secretion [26], [27]. In the stage-1 study, we selected reported SNPs of *IDE* (rs4646953, rs4646958, rs1887922, rs4646957 and rs2149632) but two of them failed during panel design of genotyping and were replaced by their respective tag SNPs (rs4304670 for rs4646957; rs6583813 for rs2149632). Amongst these SNPs of *IDE*, only rs6583813 showed significance in the stage-1 study and was replicated in later stages using additional cohorts. In a GWAS examining risk variants for multiple diseases in Icelanders, our group contributed to the discovery of the risk association of T2D with rs1111875 which lies in the intergenic region of the *HHEX-IDE* LD locus [28]. This SNP has now been validated in multiple cohorts albeit with inter-ethnic differences in allele frequency and effect size [3], [29], [30]. Other researchers have reported the close proximity of this SNP with highly conserved non-coding elements which may control expression of TFs [31]. The importance of this *HHEX-IDE* block was further supported by the joint effects of *TCF7L2*, *HHEX* and *IDE* on risk of T2D [17]. In the present analysis, the SNP discovered in our study, rs6583813, was located near the 3′ end of *IDE* with a moderate LD (r^2^ = 0.67 in Hong Kong Chinese) with rs1111875. Although we cannot be absolutely certain about the independent effect of rs6583813, in a recent epigenome study of human pancreatic islets, this novel SNP was found to lie within a putative regulatory element (NCBI Build 36.1 CHR10:94,199,479–94,203,011) implicated in epigenetics [21].

### Carboxypeptidase E (CPE)

Regulation of gene expression (epigenetics) is a complex process involving chromatin and histone modifications which play pivotal roles in determining cellular structure and function [20], [21], [32]. In a recent epigenetic study on islet cells, *CPE* is one of the reported genes contained in an islet-selective open chromatin [20] which encompasses various gene regulatory elements. Despite their upstream locations from the promoter and transcription start site, these elements recruit TFs to form a DNA loop to bring them into interactions with promoter to regulate gene expression [33]. Our results indicated that *CPE* variants at rs1583645 exhibited differential transcriptional activity, suggesting that they might alter gene expression via DNA-protein interactions. On bioinformatics analysis, 11 TFs were predicted to bind to either one or both variants [G/A]. While these predictions need experimental confirmation, two of these TFs, upstream stimulating factor (*USF*) which binds to the G-allele and octamer binding factor 1 (*Oct1* also known as *POU2F1*) which binds to the A-allele of *CPE*, are located within the chromosome 1q region which is the most replicable loci for T2D in multiple populations [34]. Besides, we have reported risk association of T2D in our Chinese populations with variants of *USF* and *POU2F1* [35], the latter also known to interact with histone proteins to alter chromatin organization and inflammatory responses [36].

### IAPP and insulin

In our family-based association test (FBAT) analysis, none of the SNPs of *CPE* or *IDE* showed associations with T2D, possibly due to small sample size and young age of the subjects. However, in this family-based cohort, non-diabetic subjects with high GRS had increased plasma IAPP and IAPP to insulin (IAPP:INS) ratio. In experimental studies, high IAPP, either *de novo* or compensatory, can induce ER stress and trigger apoptotic signaling pathways with increased expressions of C/EBP homologous proteins (CHOP) and caspase-3 [37]. In *in vitro* studies, we have demonstrated beta cell toxicity associated with IAPP oligomerization due to mitochondrial dysfunction and oxidative stress [9], [10].

Insulin and IAPP form complexes in secretory granules and are secreted in a fixed ratio. However, subject to different stimuli or conditions, these peptides may exhibit different kinetics and responses. Both insulin and IAPP share similar transcriptional regulators and enzymatic pathways for maturation and degradation [38], [39]. Thus, reduced *CPE* activity may lead to low insulin response with compensatory increase of pro-IAPP or alternatively, high *CPE* activity may increase IAPP production. Both scenarios can potentially lead to beta cell toxicity due to excessive oligomerization especially in the presence of reduced IAPP clearance. In experimental studies, exposure to fatty acids induced overexpression of IAPP resulting in impaired insulin secretion [40]. Thus, genetic or acquired factors which perturb activities of these processing and degrading enzymes may alter IAPP:INS ratio to increase risk of IAPP oligomerization, fibril formation, beta cell dysfunction and T2D [41]. Without measuring *CPE* and *IDE* activity, the final effects of these functional SNPs remain uncertain, although the multiple associations between GRS and risk of T2D, reduced beta cell function and increased IAPP support the functional significance of these variants and our overall hypothesis.

### Study limitations

Although the combined cohort of 9,901 subjects had over 90% power to detect at least 20% increased risk of T2D for SNPs with MAF≥0.05, our first stage study involving 459 young patients with familial T2D and 419 controls might have excluded some SNPs with low MAF or effect size resulting in type 2 error. Analysis of these SNPs in a larger sample size will be needed to ascertain their associations with risk of T2D. To overcome possible type 1 error, we performed 10,000 permutation tests to adjust for multiple comparisons. In the quantitative trait analysis, the IAPP results might be confounded by cross-reactivity of the IAPP antibody with pro-IAPP. Although the genetic and bioinformatics analysis on rs1583645 and rs6583813 of *CPE* and *IDE* support their functional significance, further studies are needed to examine their effects on IAPP, insulin and/or other substrates. Finally, effects due to adjacent variants via LD structures with stronger causal relationships cannot be excluded.

## Conclusion

In this study, we combined our understanding of the IAPP pathway with genetic analysis and used multiple cohorts to demonstrate the genotype-phenotype correlations relevant to T2D and beta cell function. Using a hypothesis driven approach, we confirmed the risk association of T2D with SNPs in *CPE* and *IDE*. In non-diabetic subjects, these risk variants were associated with reduced beta cell function, increased IAPP levels and IAPP:INS ratio. Bioinformatics and functional analyses suggested that these SNPs are located within regulatory sites for DNA-protein binding. Although the effect size of these SNPs averaged 15–20%, they can be found in 5% to 50% of the population. It has been estimated that for complex diseases such as T2D, 50% of population attribution risks can be explained by 20 or fewer susceptibility genes with an effect size of 10–20% [42]. Taken together, our findings support the important roles of IAPP processing and degrading enzymes in T2D and that a multi-staged approach using tag SNPs of candidate genes within a biological pathway may discover novel variants to identify high risk subjects for T2D.

## Methods

### Recruitment of samples

#### Stage-1 study

In the stage-1 study, we selected 459 T2D subjects diagnosed before 40 year-old who had at least one affected first degree relative from the Hong Kong Diabetes Registry (HKDR) [43]. None of these patients had clinical or autoimmune type 1 diabetes, defined as history of ketoacidosis or continuous requirement of insulin within 1 year of diagnosis with or without autoimmune antibodies. Another 419 control subjects with normal glucose tolerance (NGT) [fasting plasma glucose (FPG)<6.1 mmol/L] and no family history of diabetes were recruited from community-based health screening programs [44].

### De novo and in silico replication

In the stage-2 study, we included case-control cohorts consisting of 3,564 Hong Kong Chinese 3,388 Chinese from Shanghai, 1,393 Koreans and 1,150 Japanese [3], [45], [46]. We also performed the FBAT analysis in 472 subjects recruited from the Hong Kong Diabetes in Family Study [47]. All participants were recruited as part of a diabetes gene discovery program in respective countries. In the stage-3 study, we performed *in silico* replication in 2 GWAS conducted in Singaporean and European populatioins including 3,955 Chinese (2,010 cases, 1,945 controls), 2,034 Malays (794 cases, 1,240 controls) and 2,146 Indians (977 cases, 1,169 controls) and 47,117 Europeans (8,130 cases and 38,987 controls) (Text S1, Table S1 and S2).

### Tag SNP selection

Using the HapMap Phase II database for Han Chinese from Beijing (www.hapmap.org), all SNPs with MAF≥0.05 in six candidate genes with ∼2 kb flanking regions were selected. Their pair-wise LD was estimated in terms of r^2^ by Haploview v 4.0RC2 [48]. Under a pair-wise tagging mode with r^2^≥0.8, 82 tag SNPs were selected. Together with 7 SNPs reported to be associated with T2D and/or related traits (rs4646953, rs4646958, rs1887922, rs4646957 and rs2149632 in *IDE*; rs2808661 and rs6689429 in *APCS*) [15], [27], [49], 89 SNPs were selected in the stage-1 study for genotyping in 459 cases and 419 controls. Nominally significant SNPs for T2D were replicated in stage-2 and stage-3 studies.

### Genotyping

In the stage-1 study, all SNPs were genotyped using the matrix-assisted laser desorption ionization-time of flight (MALDI-TOF) MassARRAY System (Sequenom, San Diego, CA) at the Genome Research Center at the University of Hong Kong or Genome Quebec Innovation Center at the McGill University. Each of 96 well plates contained negative controls and duplicate samples for QC. Only SNPs with genotyping call rates≥0.8, MAF≥0.05 and exhibiting no departure from HWE in control subjects (*P*>0.001) were included for analysis. Stage-2 genotyping was performed using either Sequenom's MassARRAY System (Sequenom, San Diego, CA) or the MGB TaqMan Assay (Applied Biosystems, Foster City, CA, USA).

### Clinical assessment and metabolic profiling

All patients enrolled in the HKDR [43] underwent structured assessments modified from the European Diabcare protocol [50]. In brief, the HKDR was established in 1995 and enrolls 30–50 ambulatory diabetic patients per week. Patients were referred by general practitioners and internists from community and hospital-based clinics or were discharged from the Prince of Wales Hospital or other regional hospitals. All patients underwent a comprehensive diabetes assessment with documentation of detailed phenotypes and clinical outcomes to form the HKDR. All control subjects underwent detailed clinical examination. A subset (N = 302) of control subjects underwent 75g oral glucose tolerance tests (OGTT) and blood samples were collected at multiple time-points for plasma glucose (PG) and insulin measurements. PG assayed enzymatically using the Roche Modular Analytics system (Roche Diagnostics GmbH, Mannheim, Germany). Insulin was assayed using the enzyme-linked immunosorbent assays (DakoCytomation, Cambridgeshire, UK). The precision of these assays was within that specified by the manufacturer. In the family-based cohort involving 472 subjects (285 cases, 187 controls), a random subcohort of 85 subjects with normal glucose tolerance had measurement of fasting plasma IAPP determined in the laboratory of Professor Garth JS Cooper using a radioimmunoassay method with an inter-assay coefficient of variation (CV) of 3.5% [51].

### Calculation

The AUC of PG and insulin during OGTT was calculated by the trapezoid rule. Insulin resistance (HOMA-IR) was calculated by the equation of [fasting insulin (mU/l) × fasting PG (mmol/l) ÷22.5] while beta cell function was estimated by two algorithms: 1) HOMA-β  =  [fasting insulin (mU/l) ×20÷ (fasting PG (mmol/l)-3.5)] and 2) Stumvoll's index of beta cell function (×10^−6^)  =  [insulin AUC_30min_ (min.pmol/l) ÷ glucose AUC_30min_ (min.mmol/l)] [52].

### Bioinformatics and functional analyses

We tracked the University of California, Santa Cruz (UCSC) human genome browser (http://genome.cse.ucsc.edu/cgi-bin/hgGateway) to examine the cross-species conservation and regulatory elements including CpG islands, chromatin structure and histone modification sites within the flanking regions of rs1583645 in *CPE* and rs6583813 in *IDE* (NCBI Build 36.1 CHR4:166,517,651–166,518,151 and CHR10:94,199,669–94,200,169 respectively). We also performed TFBS prediction using the MATCH^TM^ program [53].

### Transient transfection studies

We generated two clones with identical sequences except [G/A] variants at rs1583645 of *CPE* into pGL4.23 vectors (Promega) by PCR cloning and QuickChange Site-Directed Mutagenesis Kit (Stratagene, La Jolla, CA, USA) using the following primers: forward:5′-TAA**GAGCTC(** ***Sac*** **I)**CAGACCTGATGAATTC-3′; reverse:5′-CTA**CTCGAG(** ***Xho*** **I)**TAGCTGTCTCTTTGAAC-3′; M1-5′-CCTATGAAGCCACAAACAAGTAATACAT**G**TGCCAGTAAAGTTGG-3′ and M2-5′-CCAACTTTACTGGCA**C**ATGTATTACTTGTTTGTGGCTTCATAGG-3 (Desired mutation underlined). We independently transfected these clones with *Renilla* luciferase vectors [pGL4.73(hRluc/SV40)] into HepG2 and rat INS-1E cells using Lipofectamine^TM^ 2000 (Invitrogen). Empty pGL4.23 vectors were included as reference for comparisons. Next, we detected their luciferase activities in cells with different variants in at least 3 independent experiments using the Dual-Luciferase Reporter Assay kit (Promega) in accordance to the manufacturer's instructions.

### Statistical analysis

All data were expressed as mean±SD or median (interquartile range) as appropriate. Skewed data were transformed using natural logarithms and outlier data (≥ or ≤4SD from the mean) were excluded. All statistical tests were performed by PLINK (v.1.07 http://pngu.mgh.harvard.edu/~purcell/plink), Haploview (v 4.0RC2 http://www.broad.mit.edu/mpg/haploview) or Stasticial Package for Social Sciences (vereson 15.0) for Windows (SPSS Inc., Chicago, IL, USA) unless specified otherwise. The study power in allelic models was estimated using PASS 2008 (NCSS, LLC. Kaysville, Utah). Assuming allelic models, our samples had over 90% power to detect at least 20% increased risk for T2D for SNPs with MAF of 0.1 and α of 0.05. The SNPs which passed QC were analyzed in each study cohort by the χ^2^ and logistic regression (LR) analysis under allelic, dominant and recessive models with or without adjustments. To adjust for multiple testings in stage-1 study, we also presented empirical *P* values by 10,000 permutations under the most significant models implemented by PLINK, which was used to select SNPs for replication.

Except for stage-1 experiment, 2-tailed *P* values<0.05 were considered statistically significant in allelic, dominant and/or recessive models unless specified otherwise. Risk association was expressed as OR with 95% CI. We selected the best model based on *P* values among genetic models for the meta-analysis of T2D in the combined cohort. The latter was performed by the Cochran-Mantel-Haenszel (CMH) test implemented in PLINK to estimate the combined ORs, 95% CI and significance level, using study population as a strata. Heterogeneity of ORs was assessed by the Cochran's Q statistic which was calculated as the weighted sum of squared differences among individual study effects and the pooled effect across studies. In case of significant heterogeneity (Q-statistic *P*<0.05), the effect size calculated from the model for random effects was also reported [54]. For the analysis of family-based cohort, Mendelian errors and potential genotyping errors were checked by PEDCHECK (v.1.1; http://watson.hgen.pitt.edu) and removed accordingly. We used the FBAT (v.2.0.3; http://www.biostat.harvard.edu/~fbat) based on the transmission disequilibrium test (TDT) but generalized to allow analysis in additive models of inheritance using –e option for testing the null hypothesis of “no linkage and no association”. The power was estimated by FBAT [55] assuming a disease prevalence of 10%, additive models with allelic OR of 1.2 for SNP with MAF of 0.1.

To test the joint effects of significant SNPs, we assigned a score of 1 to each risk allele to generate GRS with a maximum of 4 in combined analysis of cohorts with *de novo* genotyping. We also applied linear regression analysis to test the effects of GRS with beta cell function in subsets of control subjects with adjustment for covariates, as appropriate. For the dual luciferase reporter assays, all experiments were performed using a triplicate set-up consisting of 3 independent tests. All results were expressed as mean±SEM and Mann-Whitney *U*-test was used to compare differences between groups.

## Supporting Information

Figure S1 **Overall hypothesis: Genetic variations in IAPP encoding pathways including maturation, stabilization and degradation and might be associated with type 2 diabetes (T2D) and beta cell dysfunction through increased formation of pro-IAPP or IAPP, oligomerization and reduced clearance of IAPP.**
(DOC)

Figure S2 **Flowchart of study design.**
(DOC)

Table S1 **Clinical characteristics of the case-control cohorts from Hong Kong, Shanghai, Korea and Japan in stage-1 and 2 genetic association** **studies.**
(DOC)

Table S2 **Clinical characteristics of the case-control cohorts in stage-3** ***in silico*** **analysis.**
(DOC)

Table S3 **SNP list for data analysis in stage-1 study ranked by the effect sizes.**
(DOC)

Table S4 **Risk allele frequencies of** ***CPE*** **rs1583645 and** ***IDE*** **rs6583813 in Asian (CHB+JPK) and European (CEU) HapMap populations.**
(DOC)

Table S5 **Bioinformatics analysis of rs1583645 in** ***CPE*** **and rs6583813 in** ***IDE*** **.**
(DOC)

Table S6 **Summary of transcription factor (TF) binding sites predicted in the region of rs1583645 [G/A] with adjacent sequences.**
(DOC)

Text S1 **Description of study populations in stage-2** ***de novo*** **and stage-3** ***in silico*** **replication.**
(DOC)

Text S2 **Members of DIAGRAM Consortium.**
(DOC)
