# Supplementary material for: Genetic Associations of Type 2 Diabetes with Islet Amyloid Polypeptide Processing and Degrading Pathways in Asian Populations
Source: PLoS One. 2013 Jun 11;8(6):e62378. doi: 10.1371/journal.pone.0062378 (PMC3679113; doi:10.1371/journal.pone.0062378)
Supplement: Figure S1 — Overall hypothesis: Genetic variations in IAPP encoding pathways including maturation, stabilization and degradation and might be associated with type 2 diabetes (T2D) and beta cell dysfunction through increased formation of pro-IAPP or IAPP, oligomerization and reduced clearance of IAPP. (DOC) [file pone.0062378.s001.doc]

Pro-IAPP

Altered

IAPP:insulin ratio

IAPP oligomerisation

Processing Pathway

(*PCSK1*, *PCSK2*, *CPE*)

Degradation Pathway

(*IDE*)

Beta cell dysfunction and type 2 diabetes (T2D)

Stabilization

(*APCS*)

Islet amyloid formation

??

??

??

IAPP

degradation

products

Key:

Carboxypeptidase E (*CPE*)

Insulin degrading enzyme (*IDE*)

Islet amyloid polypeptide (*IAPP*)

Prohormone convertase 1 (*PCSK1*)

Prohormone convertase 2 (*PCSK2*)

Serum P amyloid component (*APCS*)

**Figure S1 Overall hypothesis: Genetic variations in IAPP encoding pathways including maturation, stabilization and degradation and might be associated with type 2 diabetes (T2D) and beta cell dysfunction through increased formation of pro-IAPP or IAPP, oligomerization and reduced clearance of IAPP.**
