# Supplementary material for: Genetic Associations of Type 2 Diabetes with Islet Amyloid Polypeptide Processing and Degrading Pathways in Asian Populations
Source: PLoS One. 2013 Jun 11;8(6):e62378. doi: 10.1371/journal.pone.0062378 (PMC3679113; doi:10.1371/journal.pone.0062378)
Supplement: Figure S2 — Flowchart of study design. (DOC) [file pone.0062378.s002.doc]

Six associated SNPs of *CPE*, *IDE* and *PCSK2* for T2D

*De novo* genotyping of six associated SNPs in *CPE*, *IDE* and *PCSK2* in an unrelated cohort consisted of 4,159 T2D patients and 4,864 controls from Hong Kong, Japan, Korea and Shanghai

A family-based related cohort of Hong Kong for the family-based association test (FBAT) analysis

SNP lookup in T2D GWAS of 8,135 Singaporeans

and 47,117 Europeans

Bioinfomatics and functional analyses

Joint effects analysis of rs1583645 in *CPE*

and rs6583813 in *IDE* for T2D and its related traits

Selection of tagging SNPs of 6 target genes using HapMap db

SNPs with previous indication of positive association

Functional SNPs

Linkage disequilibrium (LD) structure, minor allele frequency (MAF) ≥0.05

Unrelated case-control association study (Hong Kong Chinese)

459 young patients with type 2 diabetes (T2D) (age of diagnosis ≤40 years with positive family history) & 419 adult controls

**Stage-3 *In silico* analysis**

**Stage-2 *de novo* replication**

**Stage-1**

**Figure S2 Flowchart of study design.**
