# Supplementary material for: Genetic Associations of Type 2 Diabetes with Islet Amyloid Polypeptide Processing and Degrading Pathways in Asian Populations
Source: PLoS One. 2013 Jun 11;8(6):e62378. doi: 10.1371/journal.pone.0062378 (PMC3679113; doi:10.1371/journal.pone.0062378)
Supplement: Table S1 — Clinical characteristics of the case-control cohorts from Hong Kong, Shanghai, Korea and Japan in stage-1 and 2 genetic association studies. (DOC) [file pone.0062378.s003.doc]

|  | Stage-1 | |  | Stage-2 | | | | | | | | | | |
| --- | --- | --- | --- | --- | --- | --- | --- | --- | --- | --- | --- | --- | --- | --- |
|  | Hong Kong Chinese | |  | Hong Kong Chinese | | | | | Shanghai Chinese | | Korean | | Japanese | |
|  | Adult |  |  |  | Adult |  | Family | | Adult |  | Adult |  | Adult |  |
|  | controls | T2D |  | Adolescents | controls | T2D | Controls | T2D | controls | T2D | controls | T2D | controls | T2D |
| N | 419 | 459 |  | 984 | 994 | 1114 | 187 | 285 | 1672 | 1716 | 632 | 761 | 582 | 568 |
| Male (%) | 40 | 38 |  | 47 | 51 | 42 | 39 | 39 | 41 | 53 | 45 | 47 | 35 | 55 |
| Age (years) | 41 ± 10 | 39 ± 8 |  | 15 ± 2 | 72 ± 5 | 53 ± 14 | 45 ± 15 | 48 ± 15 | 58 ± 12 | 61 ± 13 | 65 ± 4 | 59 ± 10 | 68 ± 9 | 62 ± 10 |
| Duration of diabetes (years) | - | 8 ± 8 |  | - | - | 5 ± 6 | - | 8 ± 10 | - | 7 ± 7 | - | 9 ± 8 | - | 16 ± 10 |
| Body mass index (kg/m^2^) | 22.5 ± 3.2 | 25.8 ± 4.7 |  | 19.9 ± 3.6 | 23.2 ± 3.2 | 25.0 ± 4.1 | 27.2 ± 4.4 | 26.0 ± 4.6 | 23.6 ± 3.3 | 24.0 ± 3.5 | 23.5 ± 3.1 | 24.5 ± 2.9 | 22.4 ± 3.2 | 24.1 ± 3.7 |
| Fasting plasma glucose (mmol/l)^a^ | 4.8  (4.5 ; 5.1) | 7.9  (6.6 ; 10.7) |  | 4.7  (4.5 ; 5) | - | 8.1  (6.6 , 10.5) | 5.0  (4.6 ; 5.4) | 6.7  (5.9 ; 8.1) | 5.0  (4.6 ; 5.4) | - | 5.0  (4.5 ; 5.3) | 8.0  (6.7; 9.9) | - | - |
| Fasting plasma insulin (pmol/l)^a^ | 40  (26 ; 57) | - |  | 49  (36 ; 68) | - | - | 60  (42 ; 90) | - | 37  (23 ; 57) | - | 43  (32 ; 58) | - | - | - |

T2D = type 2 diabetes

All continuous values were shown as mean ± SD or ^a^ median (interquartile range).

**Table S1 Clinical characteristics of the case-control cohorts from Hong Kong, Shanghai, Korea and Japan in stage-1 and 2 genetic association studies**
