# Supplementary material for: Genetic Associations of Type 2 Diabetes with Islet Amyloid Polypeptide Processing and Degrading Pathways in Asian Populations
Source: PLoS One. 2013 Jun 11;8(6):e62378. doi: 10.1371/journal.pone.0062378 (PMC3679113; doi:10.1371/journal.pone.0062378)
Supplement: Table S2 — Clinical characteristics of the case-control cohorts in stage-3 in silico analysis. (DOC) [file pone.0062378.s004.doc]

**Table S2 Clinical characteristics of the case-control cohorts in stage-3 *in silico* analysis.**

|  | Chinese | | Chinese |  | |  | |  | |  | |
| --- | --- | --- | --- | --- | --- | --- | --- | --- | --- | --- | --- |
|  | (Illumina610quad) | | (Illumina1M) |  | | Malays | | Indians | | Europeans | |
|  | Controls | T2D | Controls | | T2D | Controls | T2D | Controls | T2D | Controls | T2D |
| N | 1,006 | 1,082 | 939 | | 928 | 1,240 | 794 | 1,169 | 977 | 38,987 | 8,130 |
| Male (%) | 22 | 37 | 64 | | 66 | 52 | 51 | 48 | 54 | - | - |
| Age (years) ^a^ | 64 | 65 | 47 | | 64 | 57 | 62 | 56 | 61 | - | - |
| Body mass index (kg/m^2^) ^a^ | 22.3 | 25.3 | 22.8 | | 25.4 | 25.1 | 27.8 | 25.3 | 27.1 | - | - |
| HbA_1C_ (%)^a^ | - | - | - | | - | 5.6 | 8.05 | 5.55 | 7.56 | - | - |
| Fasting plasma glucose (mmol/l)^a^ | 4.67 | - | 4.73 | | - | - | - | - | - | - | - |

T2D = type 2 diabetes.

^a^ Mean values are shown.
