# Supplementary material for: Genetic Associations of Type 2 Diabetes with Islet Amyloid Polypeptide Processing and Degrading Pathways in Asian Populations
Source: PLoS One. 2013 Jun 11;8(6):e62378. doi: 10.1371/journal.pone.0062378 (PMC3679113; doi:10.1371/journal.pone.0062378)
Supplement: Table S3 — SNP list for data analysis in stage-1 study ranked by the effect sizes. (DOC) [file pone.0062378.s005.doc]

**Table S3 SNP list for data analysis in stage-1 study ranked by the effect sizes.**

|  |  | CHR:bp in |  | Allele | Allele 1 frequency | Call | HWE *P*-value | Allelic ORs (95% CI) | *P* | *P_empirical_* | Power*_allelic_* |
| --- | --- | --- | --- | --- | --- | --- | --- | --- | --- | --- | --- |
| No | SNP | NCBI Build 36.1 | Gene | 1/2 | (non-DM controls) | rate | (non-DM controls) | for type2 DM | value | value | (α=0.05, one-sided ) |
| **1** | **rs17046561** | **CHR4:166,639,887** | ***CPE*** | **G/A** | **0.83** | **0.96** | **0.53** | **1.34(1.02-1.75)** | **0.03** | **0.03** | **0.705** |
| 2 | rs7729742 | CHR5:95,799,761 | *PCSK1* | T/A | 0.87 | 0.98 | 0.72 | 1.33(0.99-1.79) | 0.06 | 0.06 | 0.604 |
| **3** | **rs8117664** | **CHR20:17,200,516** | ***PCSK2*** | **G/T** | **0.14** | **0.97** | **0.64** | **1.32(1.02-1.72)** | **0.04** | **0.03** | **0.678** |
| **4** | **rs10021007** | **CHR4:166,590,962** | ***CPE*** | **C/A** | **0.61** | **0.97** | **1.00** | **1.30(1.06-1.58)** | **0.01** | **0.01** | **0.837** |
| **5** | **rs6841638** | **CHR4:166,589,111** | ***CPE*** | **G/T** | **0.74** | **0.97** | **0.60** | **1.30(1.04-1.62)** | **0.02** | **0.04** | **0.758** |
| **6** | **rs1583645** | **CHR4:166,517,901** | ***CPE*** | **G/A** | **0.73** | **0.90** | **0.82** | **1.26(1.00-1.57)** | **0.05** | **0.05** | **0.674** |
| 7 | rs11135457 | CHR5:95,758,524 | *PCSK1* | T/A | 0.12 | 0.97 | 0.88 | 1.26(0.96-1.67) | 0.1 | 0.11 | 0.503 |
| **8** | **rs6583813** | **CHR10:94,199,919** | ***IDE*** | **C/T** | **0.32** | **0.97** | **0.20** | **1.24(1.01-1.51)** | **0.04** | **0.04** | **0.686** |
| 9 | rs17687381 | CHR4:166,593,331 | *CPE* | C/A | 0.80 | 0.86 | 0.27 | 1.23(0.95-1.59) | 0.12 | 0.14 | 0.509 |
| 10 | rs4304670 | CHR10:94,321,119 | *IDE* | C/T | 0.23 | 0.95 | 0.09 | 1.22(0.98-1.53) | 0.08 | 0.09 | 0.559 |
| 11 | rs13126038 | CHR4:166,553,239 | *CPE* | G/T | 0.28 | 0.96 | 0.37 | 1.20(0.97-1.48) | 0.09 | 0.06 | 0.540 |
| 12 | rs6850689 | CHR4:166,635,903 | *CPE* | A/G | 0.24 | 0.92 | 0.21 | 1.20(0.96-1.51) | 0.11 | 0.09 | 0.506 |
| 13 | rs6044776 | CHR20:17,315,594 | *PCSK2* | C/T | 0.18 | 0.98 | 0.49 | 1.20(0.94-1.53) | 0.14 | 0.13 | 0.441 |
| 14 | rs1340937 | CHR20:17,240,818 | *PCSK2* | G/C | 0.21 | 0.97 | 0.41 | 1.18(0.94-1.48) | 0.16 | 0.16 | 0.420 |
| 15 | rs7091270 | CHR10:94,227,405 | *IDE* | G/T | 0.19 | 0.96 | 0.96 | 1.17(0.92-1.48) | 0.2 | 0.19 | 0.371 |
| 16 | rs6111477 | CHR20:17,200,253 | *PCSK2* | G/C | 0.50 | 0.97 | 1.00 | 1.15(0.95-1.39) | 0.15 | 0.14 | 0.427 |
| 17 | rs7692951 | CHR4:166,636,093 | *CPE* | A/G | 0.63 | 0.97 | 0.41 | 1.15(0.94-1.40) | 0.18 | 0.18 | 0.404 |
| 18 | rs271942 | CHR5:95,746,331 | *PCSK1* | C/T | 0.26 | 0.97 | 0.17 | 1.15(0.93-1.43) | 0.19 | 0.2 | 0.365 |
| 19 | rs3781239 | CHR10:94,207,777 | *IDE* | G/C | 0.89 | 0.97 | 0.09 | 1.15(0.84-1.59) | 0.37 | 0.35 | 0.223 |
| 20 | rs6850000 | CHR4:166,523,622 | *CPE* | A/G | 0.71 | 0.86 | 0.80 | 1.14(0.91-1.43) | 0.26 | 0.26 | 0.336 |
| 21 | rs17624818 | CHR4:166,523,716 | *CPE* | T/C | 0.81 | 0.97 | 0.46 | 1.13(0.88-1.44) | 0.35 | 0.34 | 0.252 |
| 22 | rs1992233 | CHR5:95,801,861 | *PCSK1* | G/T | 0.55 | 0.96 | 0.66 | 1.12(0.92-1.36) | 0.26 | 0.26 | 0.320 |
| 23 | rs2053277 | CHR4:166,546,781 | *CPE* | T/C | 0.11 | 0.94 | 0.59 | 1.11(0.82-1.50) | 0.5 | 0.53 | 0.170 |
| 24 | rs2072960 | CHR20:17,399,285 | *PCSK2* | A/G | 0.58 | 0.95 | 0.23 | 1.09(0.89-1.32) | 0.41 | 0.41 | 0.225 |
| 25 | rs7441954 | CHR4:166,615,409 | *CPE* | A/T | 0.33 | 0.97 | 0.35 | 1.08(0.88-1.31) | 0.48 | 0.53 | 0.189 |
| 26 | rs1446965 | CHR1:157,829,390 | *APCS* | G/A | 0.45 | 0.97 | 0.95 | 1.06(0.88-1.29) | 0.51 | 0.56 | 0.150 |
| 27 | rs2072959 | CHR20:17,399,220 | *PCSK2* | G/A | 0.64 | 0.97 | 0.02 | 1.06(0.87-1.29) | 0.58 | 0.59 | 0.145 |
| 28 | rs4933231 | CHR10:94,196,162 | *IDE* | C/T | 0.15 | 0.96 | 0.14 | 1.05(0.80-1.37) | 0.73 | 0.69 | 0.101 |
| 29 | rs6136105 | CHR20:17,399,801 | *PCSK2* | G/T | 0.13 | 0.95 | 0.54 | 1.05(0.80-1.39) | 0.72 | 0.7 | 0.097 |
| 30 | rs17085908 | CHR5:95,807,831 | *PCSK1* | C/T | 0.30 | 0.96 | 0.18 | 1.04(0.85-1.28) | 0.7 | 0.69 | 0.102 |
| 31 | rs1446966 | CHR1:157,830,107 | *APCS* | G/A | 0.18 | 1.00 | 0.41 | 1.03(0.80-1.31) | 0.84 | 0.83 | 0.080 |
| 32 | rs6810964 | CHR4:166,542,756 | *CPE* | G/A | 0.46 | 0.97 | 0.46 | 1.03(0.85-1.25) | 0.77 | 0.76 | 0.091 |
| 33 | rs6849361 | CHR4:166,553,380 | *CPE* | T/C | 0.71 | 0.97 | 0.78 | 1.03(0.84-1.27) | 0.77 | 0.82 | 0.086 |
| 34 | rs1010291 | CHR20:17,374,687 | *PCSK2* | C/T | 0.34 | 0.97 | 0.68 | 1.02(0.84-1.25) | 0.82 | 0.8 | 0.074 |
| 35 | rs3792744 | CHR5:95,768,069 | *PCSK1* | C/A | 0.84 | 0.97 | 0.65 | 1.02(0.78-1.33) | 0.89 | 0.87 | 0.068 |
| 36 | rs3792747 | CHR5:95,793,676 | *PCSK1* | T/C | 0.86 | 0.97 | <0.01 | 1.02(0.78-1.34) | 0.89 | 0.91 | 0.067 |
| 37 | rs6136101 | CHR20:17,381,800 | *PCSK2* | T/A | 0.81 | 0.91 | 0.58 | 1.02(0.80-1.30) | 0.89 | 0.88 | 0.069 |
| 38 | rs9308104 | CHR4:166,529,209 | *CPE* | A/G | 0.56 | 0.91 | 0.01 | 1.02(0.84-1.24) | 0.83 | 0.86 | 0.075 |
| 39 | rs2322291 | CHR4:166,530,129 | *CPE* | A/G | 0.43 | 0.97 | 0.16 | 1.02(0.84-1.23) | 0.85 | 0.86 | 0.075 |
| 40 | rs4690818 | CHR4:166,574,572 | *CPE* | T/C | 0.81 | 0.97 | 0.21 | 1.02(0.80-1.31) | 0.87 | 0.8 | 0.069 |
| 41 | rs2209972 | CHR10:94,169,008 | *IDE* | C/T | 0.23 | 0.95 | 0.89 | 1.01(0.86-1.37) | 0.43 | 0.42 | 0.060 |
| 42 | rs16842306 | CHR1:157,832,008 | *APCS* | T/C | 0.91 | 0.97 | 0.32 | 1.00(0.72-1.39) | 1 | 0.97 | 0.050 |
| 43 | rs9999764 | CHR4:166,521,290 | *CPE* | G/A | 0.65 | 0.84 | 0.07 | 1.00(0.81-1.24) | 0.99 | 0.99 | 0.050 |
| 44 | rs2276931 | CHR4:166,627,967 | *CPE* | C/T | 0.18 | 0.97 | 0.09 | 0.99(0.77-1.27) | 0.95 | 0.93 | 0.059 |
| 45 | rs1887922 | CHR10:94,214,145 | *IDE* | C/T | 0.09 | 0.96 | 0.23 | 0.99(0.70-1.39) | 0.95 | 0.97 | 0.056 |
| 46 | rs1037144 | CHR1:157,834,472 | *APCS* | G/A | 0.85 | 0.96 | 0.28 | 0.99(0.76-1.29) | 0.95 | 0.92 | 0.058 |
| 47 | rs2164864 | CHR4:166,543,877 | *CPE* | C/T | 0.36 | 0.96 | 0.99 | 0.99(0.81-1.21) | 0.92 | 0.93 | 0.061 |
| 48 | rs3762986 | CHR5:95,796,618 | *PCSK1* | T/C | 0.56 | 0.96 | 0.65 | 0.98(0.81-1.19) | 0.84 | 0.85 | 0.076 |
| 49 | rs155979 | CHR5:95,795,654 | *PCSK1* | G/C | 0.76 | 0.96 | 0.26 | 0.98(0.78-1.22) | 0.86 | 0.91 | 0.072 |
| 50 | rs6689429 | CHR1:157,824,145 | *APCS* | G/A | 0.81 | 0.97 | 0.08 | 0.98(0.77-1.24) | 0.86 | 0.83 | 0.070 |
| 51 | rs156001 | CHR5:95,810,027 | *PCSK1* | G/T | 0.53 | 0.96 | 0.38 | 0.97(0.80-1.17) | 0.73 | 0.73 | 0.092 |
| 52 | rs12480205 | CHR20:17,274,948 | *PCSK2* | A/G | 0.26 | 0.95 | 0.99 | 0.97(0.78-1.21) | 0.79 | 0.76 | 0.086 |
| 53 | rs1446973 | CHR1:157,848,561 | *APCS* | A/G | 0.42 | 0.95 | 0.78 | 0.96(0.79-1.16) | 0.65 | 0.68 | 0.111 |
| 54 | rs17502674 | CHR4:166,602,401 | *CPE* | G/C | 0.77 | 0.90 | 0.61 | 0.96(0.76-1.21) | 0.71 | 0.72 | 0.100 |
| 55 | rs11907226 | CHR20:17,410,150 | *PCSK2* | C/T | 0.81 | 0.95 | 0.70 | 0.96(0.75-1.22) | 0.73 | 0.69 | 0.095 |
| 56 | rs6080701 | CHR20:17,392,862 | *PCSK2* | C/T | 0.56 | 0.91 | 0.03 | 0.95(0.78-1.15) | 0.59 | 0.61 | 0.133 |
| 57 | rs3920552 | CHR20:17,287,545 | *PCSK2* | T/A | 0.59 | 0.88 | 0.17 | 0.95(0.77-1.16) | 0.6 | 0.62 | 0.132 |
| 58 | rs6044730 | CHR20:17,229,762 | *PCSK2* | G/A | 0.10 | 0.94 | 0.78 | 0.95(0.69-1.31) | 0.75 | 0.72 | 0.094 |
| 59 | rs6080705 | CHR20:17,401,598 | *PCSK2* | C/A | 0.60 | 0.97 | 0.73 | 0.94(0.77-1.14) | 0.5 | 0.55 | 0.157 |
| 60 | rs6235 | CHR5:95,754,654 | *PCSK1* | C/G | 0.32 | 0.96 | 0.73 | 0.94(0.77-1.16) | 0.57 | 0.59 | 0.148 |
| 61 | rs156019 | CHR5:95,773,119 | *PCSK1* | A/T | 0.50 | 0.97 | 0.36 | 0.93(0.77-1.12) | 0.42 | 0.41 | 0.188 |
| 62 | rs1530042 | CHR4:166,609,008 | *CPE* | T/C | 0.21 | 0.93 | 0.81 | 0.93(0.74-1.19) | 0.58 | 0.62 | 0.152 |
| 63 | rs4691198 | CHR4:166,588,491 | *CPE* | C/A | 0.18 | 0.97 | 0.16 | 0.93(0.72-1.20) | 0.57 | 0.56 | 0.142 |
| 64 | rs4690821 | CHR4:166,625,034 | *CPE* | T/C | 0.17 | 0.96 | 0.10 | 0.93(0.72-1.20) | 0.58 | 0.55 | 0.139 |
| 65 | rs1370687 | CHR4:166,609,805 | *CPE* | C/T | 0.19 | 0.97 | 0.89 | 0.92(0.72-1.17) | 0.49 | 0.52 | 0.167 |
| 66 | rs1438119 | CHR4:166,567,796 | *CPE* | C/T | 0.62 | 0.96 | 0.15 | 0.91(0.75-1.11) | 0.38 | 0.37 | 0.247 |
| 67 | rs6136041 | CHR20:17,195,207 | *PCSK2* | G/A | 0.38 | 0.97 | 0.10 | 0.91(0.74-1.10) | 0.33 | 0.28 | 0.244 |
| 68 | rs3775311 | CHR4:166,606,394 | *CPE* | C/T | 0.95 | 0.86 | 0.05 | 0.91(0.59-1.41) | 0.68 | 0.68 | 0.117 |
| 69 | rs3790336 | CHR20:17,407,928 | *PCSK2* | A/T | 0.34 | 0.97 | 0.70 | 0.90(0.73-1.10) | 0.31 | 0.31 | 0.270 |
| 70 | rs6044751 | CHR20:17,279,333 | *PCSK2* | G/A | 0.84 | 0.96 | 0.53 | 0.90(0.70-1.17) | 0.43 | 0.42 | 0.204 |
| 71 | rs13148844 | CHR4:166,522,580 | *CPE* | C/G | 0.64 | 0.95 | 0.42 | 0.89(0.73-1.09) | 0.26 | 0.26 | 0.321 |
| 72 | rs1370682 | CHR4:166,515,829 | *CPE* | G/A | 0.66 | 0.96 | 0.86 | 0.89(0.72-1.08) | 0.24 | 0.24 | 0.315 |
| 73 | rs16998899 | CHR20:17,209,305 | *PCSK2* | C/A | 0.22 | 0.97 | 0.74 | 0.89(0.71-1.13) | 0.34 | 0.32 | 0.260 |
| 74 | rs2275221 | CHR10:94,286,967 | *IDE* | C/T | 0.94 | 0.97 | 0.78 | 0.88(0.59-1.31) | 0.53 | 0.52 | 0.155 |
| 75 | rs1832197 | CHR10:94,288,311 | *IDE* | G/A | 0.83 | 0.97 | 0.09 | 0.87(0.68-1.11) | 0.26 | 0.25 | 0.301 |
| 76 | rs12624727 | CHR20:17,173,515 | *PCSK2* | G/A | 0.14 | 0.94 | 0.96 | 0.87(0.66-1.16) | 0.36 | 0.38 | 0.257 |
| 77 | rs6111527 | CHR20:17,316,967 | *PCSK2* | G/A | 0.30 | 0.97 | 0.68 | 0.86(0.69-1.06) | 0.15 | 0.13 | 0.412 |
| 78 | rs6835167 | CHR4:166,629,821 | *CPE* | C/T | 0.17 | 0.96 | 0.87 | 0.86(0.66-1.11) | 0.25 | 0.26 | 0.317 |
| 79 | rs1446972 | CHR1:157,849,970 | *APCS* | A/G | 0.92 | 0.96 | 0.71 | 0.86(0.61-1.22) | 0.4 | 0.39 | 0.218 |
| 80 | rs4646958 | CHR10:94,204,339 | *IDE* | T/A | 0.90 | 0.96 | 0.77 | 0.84(0.62-1.14) | 0.26 | 0.24 | 0.308 |
| 81 | rs6811043 | CHR4:166,542,507 | *CPE* | A/G | 0.19 | 0.94 | 0.51 | 0.82(0.63-1.05) | 0.12 | 0.12 | 0.469 |
| 82 | rs2269012 | CHR20:17,363,959 | *PCSK2* | C/T | 0.86 | 0.97 | 0.25 | 0.81(0.62-1.06) | 0.12 | 0.11 | 0.474 |
| 83 | rs6136050 | CHR20:17,210,715 | *PCSK2* | G/A | 0.91 | 0.96 | 0.31 | 0.77(0.56-1.06) | 0.11 | 0.11 | 0.500 |
| 84 | rs11698919 | CHR20:17,322,513 | *PCSK2* | G/A | 0.08 | 0.97 | 0.38 | 0.77(0.54-1.12) | 0.17 | 0.22 | 0.412 |
| 85 | rs1015777 | CHR20:17,227,771 | *PCSK2* | T/A | 0.90 | 0.97 | 0.75 | 0.76(0.56-1.02) | 0.07 | 0.06 | 0.576 |

SNPs were sorted by the effect size for type 2 diabetes (T2D). Significant SNPs for T2D (*P*≤0.05) under allelic models were shown in bold. *P_empirical_* values were presented by 10,000 permutations under the best model of genetic models implemented in PLINK for multiple test correction.
