# Supplementary material for: Genetic Associations of Type 2 Diabetes with Islet Amyloid Polypeptide Processing and Degrading Pathways in Asian Populations
Source: PLoS One. 2013 Jun 11;8(6):e62378. doi: 10.1371/journal.pone.0062378 (PMC3679113; doi:10.1371/journal.pone.0062378)
Supplement: Table S4 — Risk allele frequencies of CPE rs1583645 and IDE rs6583813 in Asian (CHB+JPK) and European (CEU) HapMap populations. (DOC) [file pone.0062378.s006.doc]

**Table S4 Risk allele frequencies of *CPE* rs1583645 and *IDE* rs6583813 in Asian (CHB+JPK) and European (CEU) HapMap populations.**

|  |  | CHR:bp in |  | Risk allele frequency | |
| --- | --- | --- | --- | --- | --- |
| SNP | Gene | NCBI Build 36.1 | Alleles^a^ | Asian (CHB+JPK)  HapMap population | European (CEU)  HapMap population |
| rs1583645 | *CPE* | CHR4:166,517,901 | **G**/A | 0.87 | 0.51 |
| rs6583813 | *IDE* | CHR10:94,199,919 | **C**/T | 0.39 | 0.68 |

^a^Risk alleles were underlined.
