# Supplementary material for: Genetic Associations of Type 2 Diabetes with Islet Amyloid Polypeptide Processing and Degrading Pathways in Asian Populations
Source: PLoS One. 2013 Jun 11;8(6):e62378. doi: 10.1371/journal.pone.0062378 (PMC3679113; doi:10.1371/journal.pone.0062378)
Supplement: Table S5 — Bioinformatics analysis of rs1583645 in CPE and rs6583813 in IDE . (DOC) [file pone.0062378.s007.doc]

**Table S5 Bioinformatics analysis of rs1583645 in *CPE* and rs6583813 in *IDE*.**

| Gene | SNP | CHR:bp in  NCBI Build 36.1 | Alleles | Mean GC% (± 250bp) | CpG island | Histone modifications | Islet FAIRE^a^ peaks | Islet DHS^b^ peaks | miRBase |
| --- | --- | --- | --- | --- | --- | --- | --- | --- | --- |
| *CPE* | rs1583645 | CHR4:166,517,901 | G/A | 33.30% | A CpG island located ~1.5kbp downstream from rs1583645 has a size of 693 bp ([chr4:166519426-166520118](http://genome.ucsc.edu/cgi-bin/hgTracks?hgsid=220684859&db=hg18&position=chr4%3A166519426-166520118)) with 20.5% CpG and 68% C/G residues. The observed to expected ratio of CpG is 0.89. | It resides in H3K9ac for HepG2 and H3K27me3 for various cell types^c^. | A FAIRE peak (chr4.5347) with 0.068 signal value located 229bp away from the rs1583645. | Two DHS peaks (chr4.4516 and 4517) with signal values of 0.162 and 0.034 located at 1.5kbp and 2.5kbp away from the rs1583645. | hsa-mir-578:  ~9kbp away from the rs1583645 |
|  |  |  |  |  |  |  |  |  |  |
| *IDE* | rs6583813 | [CHR10:94,199,919](http://genome.ucsc.edu/cgi-bin/hgTracks?hgsid=220684859&db=hg18&position=chr10%3A94199919-94199919) | C/T | 39.50% | Nil | It locates in H3K36me3 for multiple cells. | A FAIRE peak (chr10.2750) with 0.07 signal value at 15kbp upstream region from the rs6583813. | A DHS peak (chr10.4439) with signal value of 0.051 near 2kbp from the rs1583645. | Nil |
| Reference |  |  |  |  |  | [1] | [2] | [1] |  |
|  |  |  |  |  |  |  |  |  |  |

**Abbreviations:**

^a^FAIRE: Formaldehyde assisted isolation of regulatory elements, a technique to isolate DNA elements with open chromatins;

^b^DHS: DNaseI hypersensitive

^c^GM12878; H1-hESC; HSMM; HUVEC; K562; NHEK and NHLF cells.

Reference:

1. Stitzel ML, Sethupathy P, Pearson DS, Chines PS, Song L, et al. (2010) Global epigenomic analysis of primary human pancreatic islets provides insights into type 2 diabetes susceptibility loci. Cell Metab 12: 443-455.

2. Giresi PG, Kim J, McDaniell RM, Iyer VR, Lieb JD (2007) FAIRE (Formaldehyde-Assisted Isolation of Regulatory Elements) isolates active regulatory elements from human chromatin. Genome Res 17: 877-885.
