# Supplementary material for: Genetic Associations of Type 2 Diabetes with Islet Amyloid Polypeptide Processing and Degrading Pathways in Asian Populations
Source: PLoS One. 2013 Jun 11;8(6):e62378. doi: 10.1371/journal.pone.0062378 (PMC3679113; doi:10.1371/journal.pone.0062378)
Supplement: Table S6 — Summary of transcription factor (TF) binding sites predicted in the region of rs1583645 [G/A] with adjacent sequences. (DOC) [file pone.0062378.s008.doc]

**Table S6 Summary of transcription factor (TF) binding sites predicted in the region of rs1583645 [G/A] with adjacent sequences.**

| Polymorphism of rs1583645 | Factor ID | Factor Name | Known functions |
| --- | --- | --- | --- |
| A-allele specific | Oct1 | Octamer binding factor 1 | Prostate development, cancer progression and inflammation |
|  | Hand1/E47 | Heart and neural crest derivatives expressed 1/E47 | - |
| G-allele specific | E47 | E47 | Regulation of immunoglobulin gene expression |
|  | USF | Upstream stimulating factor | Glucose and lipid metabolism |
|  | MyoD | Myoblast determination gene product | Muscle cell development |
|  | N-Myc | N-Myc | V-Myc myelocytomatosis viral related oncogene, neuroblastoma derived |
|  | c-Myc/Max | MYC associated factor X | Cell proliferation, differentiation and apoptosis |
|  | AREB6 | zinc finger E-box binding homeobox 1 | Cancer |
| Both alleles | S8 | S8 | - |
|  | CCAAT | Cellular and viral CCAAT box | - |
|  | NF-Y | Nuclear factor Y (Y-box binding factor) | - |
