# Supplementary material for: Genetic Associations of Type 2 Diabetes with Islet Amyloid Polypeptide Processing and Degrading Pathways in Asian Populations
Source: PLoS One. 2013 Jun 11;8(6):e62378. doi: 10.1371/journal.pone.0062378 (PMC3679113; doi:10.1371/journal.pone.0062378)
Supplement: Text S1 — Description of study populations in stage-2 de novo and stage-3 in silico replication. (DOC) [file pone.0062378.s009.doc]

**Supplementary Information**

**Description of study populations in stage-2 *de novo* and stage-3 *in silico* replication**

***Stage-2 de novo genotyping and GWAS in silico analysis***

***Hong Kong Chinese subjects***

We used an unrelated case-control cohort and a family-based cohort to validate putative signals from stage-1 study. The former consisted of 952 T2D patients consecutively recruited from the Hong Kong Diabetes Registry [1] irrespective of age at diagnosis and 162 unrelated T2D patients (age 42±12 years, 39% male; 115 with early onset familial T2D) recruited from the Hong Kong Family Diabetes Study (HKFDS) [2]. Briefly, all cases had T2D according to the World Health Organization (WHO) criteria [3]. None of these patients had clinical or autoimmune T1D. For the control subjects, we selected 984 adolescents (age 15±2 years, 47% male) from a community-based school survey [4] and 994 healthy elderly (age 72±5 years, 51% male) from a community-based bone health study [5]. None of these subjects had past medical history or treated with regular medications.

For the FBAT analysis, we recruited 472 related subjects including 162 T2D probands and 310 first degree relatives from HKFDS [2]. In these 472 related subjects, 285 had T2D including the probands. This study was approved by the Clinical Research Ethics Committee of the Chinese University of Hong Kong. Written informed consent was obtained from each participant who donated their DNA for research purpose.

***Eastern Chinese subjects***

All subjects were of north-eastern Chinese Han ancestry and resided in Shanghai, comprising 1,716 T2D patients and 1,672 controls with detailed information described elsewhere [6]. Briefly, all cases had T2D according to the WHO criteria [3]. The inclusion criteria for control subjects were 1) age>40 years, 2) normal glucose tolerance (NGT) during a 75-gram oral glucose tolerance test (OGTT) (fasting PG<6.1 mmol/L and 2-h PG<7.8 mmol/L), and 3) absence of family history based on standard questionnaire. This study was approved by the institutional review board of the Shanghai Jiao Tong University Affiliated Sixth People’s Hospital. Written informed consent was obtained from each participant.

***Korean subjects***

The Korea Seoul National University Hospital (SNUH) case-control population consisted of 761 unrelated T2D patients registered at the Diabetes Clinic of SNUH and 632 control subjects described elsewhere [7]. Diagnosis of T2D was based on the WHO criteria [3]. Patients positive for glutamic acid decarboxylase (GAD) antibodies were excluded. Control subjects were selected based on the following criteria: age≥60 years, no known history of diabetes or affected first degree relatives, fasting PG<6.1 mmol/L and HbA_1c_<5.8%. The Institutional Review Board of the Clinical Research Institute in SNUH approved the study protocol, and informed consent for genetic analysis was obtained from each subject.

***Japanese subjects***

A total of 568 unrelated Japanese T2D patients and 582 healthy control subjects were recruited from the outpatient clinic of the Wakayama Medical University Hospital with reported clinical characteristics [8]. All T2D patients were diagnosed by the WHO criteria [3]. Patients positive for anti-GAD antibody and/or treated with insulin within 3 years of diagnosis were excluded. All control subjects were at least 50-year-old and had HbA_1c_<5.6%. All participants gave written informed consent with approval by the ethics committee of the Wakayama Medical University.

***Singapore Chinese, Malay and Indian populations***

The Singapore case-control study contained subjects from four sources: 1) 1998 Singapore National Health Survey (NHS98); 2) Singapore Malay Eye Study (SiMES); 3) Singapore Indian Eye Study (SINDI) and 4) Singapore Diabetes Cohorts Study (SDCS) [9].

In the NHS98 cohort, subjects with FPG<6.0 and 2 hour post-challenge glucose (2HPG) <7.0 mmol/l were defined as NGT. Subjects with FPG ≥6.0 and <7.0 mmol/l, and 2HPG≥ 7.0 and <7.8 mmol/l, were defined as having impaired fasting glycemia (IFG). Subjects with FPG≥7.0 mmol/l, and 2HPG ≥7.8 and <11.1 mmol/l, were defined as impaired glucose tolerance (IGT). A total of 838 IFG/IGT subjects were excluded, leaving 3,032 control subjects with NGT (2,196 Chinese, 472 Malays, and 364 Indians) available for selection.

In the NHS98 and SDCS cohorts, cases were defined by 1) a reported history of T2D; 2) FPG ≥7.0 mmol/l; or 3) 2H PG≥11.1 mmol/l. A total of 453 NHS98 cases (224 Chinese, 113 Malays, and 116 Indians) and 1,703 SDCS cases (1,317 Chinese, 256 Malays, and 130 Indians) were available for selection.

In the SiMES cohort, a total of 3,280 subjects were available for selection. Subjects with non-fasting PG<11.1 mmol/l and HbA_1c_<6.0% (2SD above the mean for the nondiabetic population) were defined as controls. Subjects with a reported history of T2D or HbA_1c_>6.5% were defined as cases [9] .

In the SINDI cohort, a total of 3,400 subjects were available for selection. Subjects without history of diabetes and HbA_1c_<6.0% were defined as controls. Subjects with a reported history of T2D or HbA_1c_>6.5% were defined as cases [9] .

From these four sources, we included 2,010 T2D cases and 1,945 NGT controls of Chinese ancestry, 794 T2D cases and 1,240 NGT controls of Malaysian ancestry, and 977 T2D cases and 1,169 NGT controls, for analysis. The clinical details of the study cohorts were shown in Table S2.

***DIAGRAM+ Study***

The DIAGRAM+ study comprised 8,130 cases and 38,987 controls of European ancestry recruited from 8 GWAS studies: 1) Diabetes Gene Discovery Group (DGDG), 2) deCODE, 3) Diabetes Genetics Initiative (DGI), 4) European Special Population Network (EUROSPAN), 5) Finland-United States Investigation of NIDDM Genetics (FUSION), 6) Cooperative Health Research in the Region of Augsburg, Southern Germany (KORAgen), 7) Rotterdam Study, 8) Wellcome Trust Case Control Consortium/ United Kingdom Type 2 Diabetes Genetics (WTCCC/UKT2D). T2D and controls were defined by study-based criteria and specified elsewhere [10] .

**References**

1. Chan JC, So W, Ma RC, Tong PC, Wong R, et al. (2011) The Complexity of Vascular and Non-Vascular Complications of Diabetes: The Hong Kong Diabetes Registry. Curr Cardiovasc Risk Rep 5: 230-239.

2. Li JK, Ng MC, So WY, Chiu CK, Ozaki R, et al. (2006) Phenotypic and genetic clustering of diabetes and metabolic syndrome in Chinese families with type 2 diabetes mellitus. Diabetes Metab Res Rev 22: 46-52.

3. Alberti KG, Zimmet PZ (1998) Definition, diagnosis and classification of diabetes mellitus and its complications. Part 1: diagnosis and classification of diabetes mellitus provisional report of a WHO consultation. Diabet Med 15: 539-553.

4. Ozaki R, Qiao Q, Wong GW, Chan MH, So WY, et al. (2007) Overweight, family history of diabetes and attending schools of lower academic grading are independent predictors for metabolic syndrome in Hong Kong Chinese adolescents. Arch Dis Child 92: 224-228.

5. Woo J, Kwok T, Leung J, Tang N (2008) Dietary intake, blood pressure and osteoporosis. J Hum Hypertens 23: 451-455.

6. Jia WP, Pang C, Chen L, Bao YQ, Lu JX, et al. (2007) Epidemiological characteristics of diabetes mellitus and impaired glucose regulation in a Chinese adult population: the Shanghai Diabetes Studies, a cross-sectional 3-year follow-up study in Shanghai urban communities. Diabetologia 50: 286-292.

7. Kwak SH, Cho YM, Moon MK, Kim JH, Park BL, et al. (2008) Association of polymorphisms in the insulin-degrading enzyme gene with type 2 diabetes in the Korean population. Diabetes Res Clin Pract 79: 284-290.

8. Furukawa Y, Shimada T, Furuta H, Matsuno S, Kusuyama A, et al. (2008) Polymorphisms in the IDE-KIF11-HHEX gene locus are reproducibly associated with type 2 diabetes in a Japanese population. J Clin Endocrinol Metab 93: 310-314.

9. Sim X, Ong RT, Suo C, Tay WT, Liu J, et al. (2011) Transferability of type 2 diabetes implicated loci in multi-ethnic cohorts from Southeast Asia. PLoS Genet 7: e1001363.

10. Voight BF, Scott LJ, Steinthorsdottir V, Morris AP, Dina C, et al. (2010) Twelve type 2 diabetes susceptibility loci identified through large-scale association analysis. Nat Genet 42: 579-589.
