# Supplementary material for: Genetic Associations of Type 2 Diabetes with Islet Amyloid Polypeptide Processing and Degrading Pathways in Asian Populations
Source: PLoS One. 2013 Jun 11;8(6):e62378. doi: 10.1371/journal.pone.0062378 (PMC3679113; doi:10.1371/journal.pone.0062378)
Supplement: Text S2 — Members of DIAGRAM Consortium. (DOC) [file pone.0062378.s010.doc]

**MEMBERS OF THE DIAGRAM+ CONSORTIUM**

Benjamin F Voight^1,2,3^, Laura J Scott^4^, Valgerdur Steinthorsdottir^5^, Andrew P Morris^6^, Christian Dina^7,8^, Ryan P Welch^9^, Eleftheria Zeggini^6,10^, Cornelia Huth^11,12^, Yurii S Aulchenko^13^, Gudmar Thorleifsson^5^, Laura J McCulloch^14^, Teresa Ferreira^6^, Harald Grallert^11,12^, Najaf Amin^13^, Guanming Wu^15^, Cristen J Willer^4^, Soumya Raychaudhuri^1,2,16^, Steve A McCarroll^1,17^, Claudia Langenberg^18^, Oliver M Hofmann^19^, Josée Dupuis^20^**,**^21^, Lu Qi^22-24^, Ayellet V Segrè^1,2,17^, Mandy van Hoek^25^, Pau Navarro^26^, Kristin Ardlie^1^, Beverley Balkau^27,28^, Rafn Benediktsson^29,30^, Amanda J Bennett^14^, Roza Blagieva^31^, Eric Boerwinkle^32^, Lori L Bonnycastle^33^, Kristina Bengtsson Boström^34^, Bert Bravenboer^35^, Suzannah Bumpstead^10^, Noël P Burtt^1^, Guillaume Charpentier^36^, Peter S Chines^33^, Marilyn Cornelis^24^, David J Couper^37^, Gabe Crawford^1^, Alex SF Doney^38,39^, Katherine S Elliott^6^, Amanda L Elliott^1,17,40^, Michael R Erdos^33^, Caroline S Fox^21,41^, Christopher S Franklin^42^, Martha Ganser^4^, Christian Gieger^11^, Niels Grarup^43^, Todd Green^1,2^, Simon Griffin^18^, Christopher J Groves^14^, Candace Guiducci^1^, Samy Hadjadj^44^, Neelam Hassanali^14^, Christian Herder^45^, Bo Isomaa^46,47^, Anne U Jackson^4^, Paul RV Johnson^48^, Torben Jørgensen^49,50^, Wen HL Kao^51,52^, Norman Klopp^11^, Augustine Kong^5^, Peter Kraft^22,23^, Johanna Kuusisto^53^, Torsten Lauritzen^54^, Man Li^51^, Aloysius Lieverse^55^, Cecilia M Lindgren^6^, Valeriya Lyssenko^56^, Michel Marre^57,58^, Thomas Meitinger^59,60^, Kristian Midthjell^61^, Mario A Morken^33^, Narisu Narisu^33^, Peter Nilsson^56^, Katharine R Owen^14^, Felicity Payne^10^, John RB Perry^62,63^, Ann-Kristin Petersen^11^, Carl Platou^61^, Christine Proença^7^, Inga Prokopenko^6,14^, Wolfgang Rathmann^64^, N William Rayner^6,14^, Neil R Robertson^6,14^, Ghislain Rocheleau^65-67^, Michael Roden^45,68^, Michael J Sampson^69^, Richa Saxena^1,2,40^, Beverley M Shields^62,63^, Peter Shrader^3,70^, Gunnar Sigurdsson^29,30^, Thomas Sparsø^43^, Klaus Strassburger^64^, Heather M Stringham^4^, Qi Sun^22,23^, Amy J Swift^33^, Barbara Thorand^11^, Jean Tichet^71^, Tiinamaija Tuomi^46,72^, Rob M van Dam^24^, Timon W van Haeften^73^, Thijs van Herpt^25,55^, Jana V van Vliet-Ostaptchouk^74^, G Bragi Walters^5^, Michael N Weedon^62,63^, Cisca Wijmenga^75^, Jacqueline Witteman^13^, Richard N Bergman^76^, Stephane Cauchi^7^, Francis S Collins^77^, Anna L Gloyn^14^, Ulf Gyllensten^78^, Torben Hansen^43,79^, Winston A Hide^19^, Graham A Hitman^80^, Albert Hofman^13^, David J Hunter^22,23^, Kristian Hveem^61,81^, Markku Laakso^53^, Karen L Mohlke^82^, Andrew D Morris^38,39^, Colin NA Palmer^38,39^, Peter P Pramstaller^83^, Igor Rudan^42,84,85^, Eric Sijbrands^25^, Lincoln D Stein^15^, Jaakko Tuomilehto^86^, Andre Uitterlinden^25^, Mark Walker^87^, Nicholas J Wareham^18^, Richard M Watanabe^76,88^, Goncalo R Abecasis^4^, Bernhard O Boehm^31^, Harry Campbell^42^, Mark J Daly^1,2^, Andrew T Hattersley^62,63^, Frank B Hu^22-24^, James B Meigs^3,70^, James S Pankow^89^, Oluf Pedersen^43,90,91^, H.-Erich Wichmann^11,12,92^, Inês Barroso^10^, Jose C Florez^1,2,3,93^, Timothy M Frayling^62,63^, Leif Groop^56,72^, Rob Sladek^65-67^, Unnur Thorsteinsdottir^5,94^, James F Wilson^42^, Thomas Illig^11^, Philippe Froguel^17,95^, Cornelia M van Duijn^13^, Kari Stefansson^5,94^, David Altshuler^1,2,3,17,40,93^, Michael Boehnke^4^, Mark I McCarthy^6,14,96^.

**Affiliations**

1. Broad Institute of Harvard and Massachusetts Institute of Technology (MIT), Cambridge, Massachusetts 02142, USA
2. Center for Human Genetic Research, Massachusetts General Hospital, 185 Cambridge Street, Boston, Massachusetts 02114, USA
3. Department of Medicine, Harvard Medical School, Boston, Massachusetts 02115, USA
4. Department of Biostatistics, University of Michigan, Ann Arbor, Michigan 48109-2029, USA
5. deCODE Genetics, 101 Reykjavik, Iceland
6. Wellcome Trust Centre for Human Genetics, University of Oxford, Oxford, OX3 7BN, UK
7. CNRS-UMR-8090, Institute of Biology and Lille 2 University, Pasteur Institute, F-59019 Lille, France
8. INSERM UMR915 CNRS ERL3147 F-44007 Nantes, France
9. Bioinformatics Program, University of Michigan, Ann Arbor MI USA 48109
10. Wellcome Trust Sanger Institute, Hinxton, CB10 1HH, UK
11. Institute of Epidemiology, Helmholtz Zentrum Muenchen, 85764 Neuherberg, Germany
12. Institute of Medical Informatics, Biometry and Epidemiology, Ludwig-Maximilians-Universität, 81377 Munich, Germany
13. Department of Epidemiology, Erasmus University Medical Center, P.O. Box 2040, 3000 CA Rotterdam, The Netherlands.
14. Oxford Centre for Diabetes, Endocrinology and Metabolism, University of Oxford, OX3 7LJ, UK
15. Ontario Institute for Cancer Research, 101 College Street, Suite 800, Toronto, Ontario M5G 0A3, Canada
16. Division of Rheumatology, Immunology and Allergy, Brigham and Women's Hospital, Harvard Medical School, Boston, Massachusetts 02115, USA
17. Department of Molecular Biology, Harvard Medical School, Boston, Massachusetts 02115, USA
18. MRC Epidemiology Unit, Institute of Metabolic Science, Addenbrooke's Hospital, Cambridge CB2 0QQ, UK
19. Department of Biostatistics, Harvard School of Public Health, Boston, Massachusetts 02115, USA
20. Department of Biostatistics, Boston University School of Public Health, Boston, Massachusetts 02118, USA
21. National Heart, Lung, and Blood Institute’s Framingham Heart Study, Framingham, Massachusetts 01702, USA
22. Department of Nutrition, Harvard School of Public Health, 665 Huntington Ave, Boston, MA 02115, USA
23. Department of Epidemiology, Harvard School of Public Health, 665 Huntington Ave, Boston, MA 02115, USA
24. Channing Laboratory, Dept. of Medicine, Brigham and Women's Hospital and Harvard Medical School, 181 Longwood Ave, Boston, MA 02115, USA
25. Department of Internal Medicine, Erasmus University Medical Centre, PO-Box 2040, 3000 CA Rotterdam, The Netherlands
26. MRC Human Genetics Unit, Institute of Genetics and Molecular Medicine, Western General Hospital, Edinburgh, EH4 2XU, UK
27. INSERM U780, F-94807 Villejuif. France
28. University Paris-Sud, F-91405 Orsay, France
29. Landspitali University Hospital, 101 Reykjavik, Iceland
30. Icelandic Heart Association, 201 Kopavogur, Iceland
31. Division of Endocrinology, Diabetes and Metabolism, Ulm University, 89081 Ulm, Germany
32. The Human Genetics Center and Institute of Molecular Medicine, University of Texas Health Science Center, Houston, Texas 77030, USA
33. National Human Genome Research Institute, National Institute of Health, Bethesda, Maryland 20892, USA
34. R&D Centre, Skaraborg Primary Care, 541 30 Skövde, Sweden
35. Department of Internal Medicine, Catharina Hospital, PO-Box 1350, 5602 ZA Eindhoven, The Netherlands
36. Endocrinology-Diabetology Unit, Corbeil-Essonnes Hospital, F-91100 Corbeil-Essonnes, France
37. Department of Biostatistics and Collaborative Studies Coordinating Center, University of North Carolina at Chapel Hill, Chapel Hill, North Carolina, 27599, USA
38. Diabetes Research Centre, Biomedical Research Institute, University of Dundee, Ninewells Hospital, Dundee DD1 9SY, UK
39. Pharmacogenomics Centre, Biomedical Research Institute, University of Dundee, Ninewells Hospital, Dundee DD1 9SY, UK
40. Department of Genetics, Harvard Medical School, Boston, Massachusetts 02115, USA
41. Division of Endocrinology, Diabetes, and Hypertension, Brigham and Women’s Hospital, Harvard Medical School, Boston, Massachusetts 02115, USA
42. Centre for Population Health Sciences, University of Edinburgh, Teviot Place, Edinburgh, EH8 9AG, UK
43. Hagedorn Research Institute, **DK-2820** Gentofte, Denmark
44. Centre Hospitalier Universitaire de Poitiers, Endocrinologie Diabetologie, CIC INSERM 0801, INSERM U927, Université de Poitiers, UFR, Médecine Pharmacie, 86021 Poitiers Cedex, France
45. Institute for Clinical Diabetology, German Diabetes Center, Leibniz Center for Diabetes Research at Heinrich Heine University Düsseldorf, 40225 Düsseldorf, Germany
46. Folkhälsan Research Center, FIN-00014 Helsinki, Finland
47. Malmska Municipal Health Center and Hospital, 68601 Jakobstad, Finland
48. Diabetes Research and Wellness Foundation Human Islet Isolation Facility and Oxford Islet Transplant Programme, University of Oxford, Old Road, Headington, Oxford, OX3 7LJ, UK
49. Research Centre for Prevention and Health, Glostrup University Hospital, DK-2600 Glostrup, Denmark
50. Faculty of Health Science, University of Copenhagen, 2200 Copenhagen, Denmark
51. Department of Epidemiology, Johns Hopkins University, Baltimore, Maryland 21287, USA
52. Department of Medicine, and Welch Center for Prevention, Epidemiology, and Clinical Research, Johns Hopkins University, Baltimore, Maryland 21287, USA
53. Department of Medicine, University of Kuopio and Kuopio University Hospital, FIN-70211 Kuopio, Finland
54. Department of General Medical Practice, University of Aarhus, DK-8000 Aarhus, Denmark
55. Department of Internal Medicine, Maxima MC, PO-Box 90052, 5600 PD Eindhoven, The Netherlands
56. Department of Clinical Sciences, Diabetes and Endocrinology Research Unit, University Hospital Malmö, Lund University, 205 02 Malmö, Sweden
57. Department of Endocrinology, Diabetology and Nutrition, Bichat-Claude Bernard University Hospital, Assistance Publique des Hôpitaux de Paris, 75870 Paris Cedex 18, France
58. INSERM U695, Université Paris 7, 75018 Paris , France
59. Institute of Human Genetics, Helmholtz Zentrum Muenchen, 85764 Neuherberg, Germany
60. Institute of Human Genetics, Klinikum rechts der Isar, Technische Universität München, 81675 Muenchen, Germany
61. Nord-Trøndelag Health Study (HUNT) Research Center, Department of Community Medicine and General Practice, Norwegian University of Science and Technology, NO-7491 Trondheim, Norway
62. Genetics of Complex Traits, Institute of Biomedical and Clinical Science, Peninsula Medical School, University of Exeter, Magdalen Road, Exeter EX1 2LU, UK
63. Diabetes Genetics, Institute of Biomedical and Clinical Science, Peninsula Medical School, University of Exeter, Barrack Road, Exeter EX2 5DW, UK
64. Institute of Biometrics and Epidemiology, German Diabetes Center, Leibniz Center for Diabetes Research at Heinrich Heine University Düsseldorf, 40225 Düsseldorf, Germany
65. Department of Human Genetics, McGill University, Montreal H3H 1P3, Canada
66. Department of Medicine, Faculty of Medicine, McGill University, Montreal, H3A 1A4, Canada
67. McGill University and Genome Quebec Innovation Centre, Montreal, H3A 1A4. Canada
68. Department of Metabolic Diseases, Heinrich Heine University Düsseldorf, 40225 Düsseldorf, Germany
69. Department of Endocrinology and Diabetes, Norfolk and Norwich University Hospital NHS Trust , Norwich, NR1 7UY, UK.
70. General Medicine Division, Massachusetts General Hospital, Boston, Massachusetts, USA
71. Institut interrégional pour la Santé (IRSA), F-37521 La Riche, France
72. Department of Medicine, Helsinki University Hospital, University of Helsinki, FIN-00290 Helsinki, Finland
73. Department of Internal Medicine, University Medical Center Utrecht, 3584 CG Utrecht,The Netherlands
74. Molecular Genetics, Medical Biology Section, Department of Pathology and Medical Biology, University Medical Center Groningen and University of Groningen, 9700 RB Groningen, The Netherlands
75. Department of Genetics, University Medical Center Groningen and University of Groningen, 9713 EX Groningen, The Netherlands
76. Department of Physiology and Biophysics, University of Southern California School of Medicine, Los Angeles, California 90033, USA
77. National Institute of Health, Bethesda, Maryland 20892, USA
78. Department of Genetics and Pathology, Rudbeck Laboratory, Uppsala University, S-751 85 Uppsala, Sweden.
79. University of Southern Denmark, DK-5230 Odense, Denmark
80. Centre for Diabetes, Barts and The London School of Medicine and Dentistry, Queen Mary University of London, London E1 2AT, UK
81. Department of Medicine, The Hospital of Levanger, N-7600 Levanger, Norway
82. Department of Genetics, University of North Carolina, Chapel Hill, North Carolina 27599, USA
83. Institute of Genetic Medicine, European Academy Bozen/Bolzano (EURAC), Viale Druso 1, 39100 Bolzano, Italy
84. Croatian Centre for Global Health, Faculty of Medicine, University of Split, Soltanska 2, 21000 Split, Croatia
85. Institute for Clinical Medical Research, University Hospital "Sestre Milosrdnice", Vinogradska 29, 10000 Zagreb, Croatia
86. Department of Chronic Disease Prevention, National Institute for Health and Welfare, Helsinki FIN-00300, Finland,
87. Diabetes Research Group, Institute of Cellular Medicine, Newcastle University, Framlington Place, Newcastle upon Tyne NE2 4HH, UK
88. Department of Preventitive Medicine, Keck Medical School, University of Southern California, Los Angeles, CA, 90089-9001, USA
89. Division of Epidemiology and Community Health, University of Minnesota, Minneapolis, Minnesota 55454, USA
90. Department of Biomedical Science, Panum, Faculty of Health Science, University of Copenhagen, 2200 Copenhagen, Denmark
91. Faculty of Health Science, University of Aarhus, DK–8000 Aarhus, Denmark
92. Klinikum Grosshadern, 81377 Munich, Germany
93. Diabetes Unit, Massachusetts General Hospital, Boston, Massachusetts 02144, USA
94. Faculty of Medicine, University of Iceland, 101 Reykjavík, Iceland
95. Genomic Medicine, Imperial College London, Hammersmith Hospital, W12 0NN, London, UK
96. Oxford National Institute for Health Research Biomedical Research Centre, Churchill Hospital, Old Road Headington, Oxford, OX3 7LJ, UK
